# The NKp44-1 Isoform Is an Activating Receptor for PDGF-DD Expressed on Natural Killer Cells

**DOI:** 10.3390/cancers18071099

**Published:** 2026-03-28

**Authors:** Alexander J. Sedgwick, Md Abdullah Al Kamran Khan, Stephanie Thuy Duong Pham, Melissa A. Edeling, Alexandra J. Corbett, Julian P. Vivian, Yaseelan Palarasah, Alexander D. Barrow

**Affiliations:** 1 Department of Microbiology and Immunology, The University of Melbourne at The Peter Doherty Institute for Infection and Immunity, Melbourne, VIC 3000, Australia; 2 Department of Molecular Medicine, Cancer and Inflammation, University of Southern Denmark, 5000 Odense, Denmark; 3 St. Vincent’s Institute of Medical Research, Melbourne, VIC 3065, Australia; 4 Faculty of Health Sciences, Australian Catholic University, Melbourne, VIC 3065, Australia

**Keywords:** natural killer cells, NKp44, platelet-derived growth factor D, immunoreceptor tyrosine-based inhibition motif, endocytosis

## Abstract

Background/Objectives: Natural killer (NK) cells are key innate lymphoid cells that restrict tumour progression by secreting proinflammatory cytokines and directly lysing malignant cells, with their activity tightly regulated by a balance of activating and inhibitory surface receptors. The natural cytotoxicity receptor NKp44 is induced on NK cells following stimulation with IL-2 or IL-15 and recognizes platelet-derived growth factor D (PDGF-DD) as a ligand. Mechanistic interpretation of NKp44 signalling upon PDGF-DD engagement is confounded by the existence of three distinct NKp44 isoforms (NKp44-1, -2, and -3), each capable of initiating divergent intracellular signalling cascades. Unlike NKp44-2 and -3, NKp44-1 encodes a cytoplasmic tyrosine residue (Y238) that conforms to a putative immunoreceptor tyrosine-based inhibition motif (ITIM) and has been reported to suppress NK cell effector functions in some contexts. However, it remains unclear whether the NKp44 isoforms are translated and expressed in NK cells, and formal evidence defining NKp44-1 signalling in response to engagement by PDGF-DD is lacking. Methods: In this study, we used C-terminal targeting monoclonal antibodies (mAbs) and a NFAT-GFP reporter system to define the expression and signalling properties of NKp44 isoforms in response to PDGF-DD. Results: We demonstrate protein expression of NKp44-1 and NKp44-2-/3 receptors in IL-2 expanded NK cells. We further show that NKp44-1 transduces activating rather than inhibitory signals when engaged by PDGF-DD ligand, albeit weaker than NKp44-3. Intriguingly, we find that Y238 is dispensable for NKp44-1 activating signalling and instead functions as a YXXΦ internalisation motif. Conclusions: Collectively, these findings provide the first evidence that the NKp44-1 and NKp44-2/3 isoforms are expressed in NK cells and establish that PDGF-DD activates signalling through NKp44-1 independently of Y238. This work lays the foundations for future studies investigating how PDGF-DD sensing by the different NKp44 isoforms shapes immune functions in different physiological and pathological contexts.

## Simple Summary

Natural killer (NK) cells play a critical role in protecting the body against infection and cancer. Their ability to recognise and eliminate malignant or infected cells is governed by many activating and inhibitory receptors expressed on their cell surface. Among these, the NKp44 receptor has been difficult to study because existing antibodies cannot distinguish individual protein isoforms—structurally similar variants of the same receptor that may have distinct functions. Notably, one isoform, NKp44-1, has been proposed to suppress NK cell activity. In this study, we developed new antibodies that can accurately detect expression of the different NKp44 isoforms on NK cells and investigated how NKp44-1 reacts when it encounters platelet-derived growth factor D (PDGF-DD), a molecule released by many cancerous and infected cells that has been shown to switch on NKp44. Our findings provide the first direct evidence of NKp44 isoform expression in NK cells and establish a foundation for understanding how the NKp44 isoforms sense PDGF-D to influence NK cell activity.

## 1. Introduction

Natural killer (NK) cells are innate cytotoxic lymphocytes that play a critical role in immune surveillance and early defence against infected or transformed cells. NK cell cytotoxicity and the secretion of proinflammatory cytokines, such as IFN-γ and TNF, are governed by the integration of signals from a surface array of activating and inhibitory receptors [1]. For example, NK cell tolerance to host tissues is maintained by inhibitory receptors such as CD94/NKG2A and the killer immunoglobulin-like receptors (KIRs), that recognise allelic variants of class I human leukocyte (HLA) antigens (HLA-I), which act as markers of a healthy “self” status [2]. Loss of HLA-I expression on target cells—a hallmark of the “missing-self” phenomenon—removes inhibitory signals [3], while concurrent upregulation of stress-induced ligands for activating receptors, as exemplified by NKG2D recognition of NKG2D ligands, such as stress-inducible MIC-A/B and the members of the retinoic acid early transcripts (RAET) family [4,5,6,7], tips the balance toward NK cell activation and evokes NK cell effector functions. Thus, understanding the myriad different activating and inhibitory receptor–ligand interactions that govern signal integration within NK cells is critical for harnessing their activity against pathogens and tumours [8,9].

NKp44, also known as Natural Cytotoxicity Receptor 2 (NCR2), was first described to trigger NK cell cytotoxicity in redirected cellular cytotoxicity assays [10]. Absent from resting NK cells, NKp44 is upregulated on the NK cell surface in complex with the DNAX accessory protein 12 (DAP12, also known as TYROBP and KARAP) following stimulation with IL-2 or IL-15 [11]. Consistent with a role as an activating receptor, NKp44 engagement triggers the phosphorylation of immunoreceptor tyrosine-based activation motifs (ITAMs: YxxL/Ix6-8YxxL, where “x” denotes any amino acid) within DAP12 leading to the recruitment of ζ-chain associated protein kinase 70 (ZAP70) or SYK [12]. Interestingly, alternative splicing of *NCR2* transcripts is predicted to generate three isoforms of NKp44, designated NKp44-1, -2, and -3 [13]. In contrast to NKp44-2 and NKp44-3, NKp44-1 encodes a longer cytoplasmic tail containing an “ILYHTV” motif conforming to a canonical immunoreceptor tyrosine-based inhibition motif (ITIM; S/I/V/LxYxxx/V/L), which also encompasses a potential endocytosis motif (Yxxϕ, where “ϕ” represents any bulky hydrophobic residue) [14,15]. Thus, NKp44-1 has the potential to be internalized and/or recruit inhibitory phosphatases, such as the SH2-domain containing phosphotyrosine phosphatase (SHP)-1 or SHP-2 or SH2 Domain-Containing Inositol 5′-Phosphatase (SHIP)-1 or -2 to reign in NK cell activation [16].

The role of the NKp44-1 receptor in NK cell immunity has been difficult to pinpoint given its ability to bind to diverse pathogen- and host-encoded ligands [17,18,19]. NKp44-1 has been reported to be preferentially expressed in poorly cytotoxic uterine NK (uNK) cells and has also been associated with poor prognosis in AML patients [20,21]. Recently, platelet-derived growth factor D (PDGF-DD) was shown to be a functional ligand for NKp44 [22]. In line with a role in triggering NK cell activation, PDGF-DD binding to NKp44 triggered NK cell cytokine secretion of IFN-γ and TNF which restricted tumour growth and spread [22]. Interestingly, PDGF-D is linked to protumorigenic signalling and poor prognosis in acute myeloid leukemia (AML) [23], bladder cancer [24,25,26,27] and low-grade glioma [28,29]. Differences in downstream signalling or surface expression of NKp44-1 relative to the NKp44-2/-3 isoforms may, therefore, be relevant in NK cell responses to cancer cells. However, whether the putative ITIM in NKp44-1 may differentially influence the downstream signalling and cellular trafficking of NKp44-PDGF-DD complexes remains unknown. Here, we developed specific mAbs to the C-terminus of the NKp44-1 and NKp44-2/3 isoforms to quantify the relative abundance of these proteins in primary NK cells, particularly as formal evidence for their expression is lacking. Moreover, using NFAT-GFP reporter cells, we set out to investigate the signalling potential of NKp44 isoforms when stimulated by PDGF-DD as ligand.

## 2. Materials and Methods

### 2.1. Bioinformatic Analysis of NKp44 Isoform Signalling Motifs

Amino acid sequences (NKp44-1: NP_004819; NKp44-2: NP_001186438; NKp44-3: NP_001186439) were obtained from NCBI and aligned using the Uniprot Align tool https://www.uniprot.org/align (accessed on 29 August 2025). For The Eukaryotic Linear Motif Resource for Functional Sites in Proteins (ELM) analysis, the amino acid sequence of NKp44-1 and NKp44-2/3 including the transmembrane domain through to the C-terminal domain was uploaded to http://elm.eu.org/ (accessed on 29 August 2025). Cytosol was selected for cell compartment, and *Homo sapiens* was selected for taxonomic context.

### 2.2. Generation of Human NKp44 Isoform-Specific mAbs

Mice were immunised with the two different peptides: NKp44-1 C-terminal peptide (ARTKISDDDDEHTL) and NKp44-2/3 C-terminal peptide (HRHFPLSHRAPGGTYGGKP). The synthetic peptides were coupled onto diphtheria toxoid via the cysteine. The immunization was performed by NMRI mice receiving two subcutaneous injections, with an interval of at least 14 days, using 25 µg of the peptides coupled with diphtheria toxoid via the end cysteine (Statens Serum Institute, Copenhagen, Denmark). The antigen was adsorbed to Al(OH)_3_ and mixed in a 1:1 ratio with Freund’s incomplete adjuvant. An intravenous boost with 12 µg of the antigens, administered with adrenalin, was performed 14 days later, followed by fusion three days after the boost. After immunization, the spleen of the mice was isolated. The isolated spleen cells and myeloma cells were fused to generate hybridoma cells. The fusion of the spleen cells and selection was performed essentially as described by Köhler and Milstein [30]; however, the SP2/0-AG14 myeloma cell line was used as fusion partner. Culture supernatants were then screened for reactivity by ELISA using microtiter plates (Nunc™ Maxisorp™, Roskilde, Denmark) coated with 2 mg/mL (diluted 1:1000) of the immunized peptides coupled to bovine serum albumin (BSA). The hybridoma cells that generated positive wells were cloned by limiting dilution. The single clones were grown in culture flasks in RPMI-1640 (Lonza, BioWhittaker^®^, Walkersville, MD, USA) supplemented with sodium pyruvate and gentamicin sulfate and containing 10% fetal bovine serum (Biowest, Lakewood Ranch, FL, USA). The development of monoclonal antibodies was conducted under approval from the Danish Animal Experiments Inspectorate (Dyreforsøgstilsynet), which operates under the Ministry of Food, Agriculture and Fisheries of Denmark. Immunizations were performed in accordance with ethical approval (approval number 2015-15-0201-00680).

### 2.3. Cell Culture

Phoenix amphotrophic [31], HEK 293T [32] and 2B4 NFAT-GFP reporter cells [33], and KHYG-1 [34] and CEM cells [35] were cultured at 37.5 °C with 5% CO_2_ in DMEM and RPMI (media preparation unit, Doherty Institute, Melbourne, VIC, Australia), respectively, supplemented with 1% GlutaMAX (Gibco, #35050-061, Grand Island, NY, USA), 1% sodium pyruvate (Sigma, #S8636-100ML, Darmstadt, Germany), 1% non-essential amino acids (Sigma, #M7145-100ML), 1% penicillin streptomycin (media preparation unit, Doherty Institute) and 10% bovine calf serum (BCS) (Sigma-Aldrich, #12138C-500ML, Lenexa, KS, USA). KHYG-1 cultures were maintained with additional 100 U/mL IL-2 (Peprotech, #200-02, Cranbury, NJ, USA). All cell lines were obtained from Barrow Laboratory stocks (Doherty Institute, Melbourne, VIC, Australia).

### 2.4. NK Cell Isolation and Expansion

Peripheral blood mononuclear cells (PBMCs) were isolated from healthy blood donors (Australian Red Cross Lifeblood) with the approval of the University of Melbourne Human Research Ethics Committee (#28023). PBMCs were purified over Ficoll-Paque density gradient (Cytiva, #17144002, Marlborough, MA, USA). CD3^−^ CD56^+^ NK cells were isolated using an EasySep^TM^ Human NK Cell Isolation Kit (Stem Cell Technologies, #17955, Vancouver, BC, Canada). NK cell purity was determined by performing flow cytometry using anti-CD3-BV421 (OKT3, BioLegend, #317344, San Diego, CA, USA) and anti-CD56-PE/Cyanine-7 (HCO56, BioLegend #318318) antibodies. NK cells were expanded using NK MACS medium (Miltenyi Biotec, #130-114-429, Bergisch Gladbach, Germany) supplemented with IL-2 for two weeks.

### 2.5. In Vitro Differentiation of “Uterine-like NK Cells”

CD3^−^ CD56^+^ NK cells were isolated from peripheral blood (described above) and cultured in RPMI supplemented with 10% AB serum (Sigma, #H5422), 5% FCS (Bovogen, #SFBS-AU, Melbourne, VIC, Australia) and 100 U/mL IL-2 with or without 10 ng/mL TGF-β (PeproTech, #100-21-2UG^®^, Cranbury, NJ, USA) for 14 days. Fifty percent of the media was replaced every two to three days. A “*uterine-like*” surface phenotype was verified using anti-CD3-AF700 (OKT3, BioLegend #317340), anti-CD56-PerCp-Cy5.5 (5.1H11, BioLegend, #362505), anti-CD16-BV510 (3G8, BioLegend #302048), anti-NKp44-AF647 (P44-8, BioLegend, #325112) anti-CD9-APC-Fire750 (H19a, BioLegend #312113), anti-CD39-BV711 (A1, BioLegend #328227), anti-CD69 (FN50, BioLegend #310929), anti-CD103-BV786 (Ber-ACT8, BD BioSciences #743654, San Diego, CA, USA), anti-CD49a-PE (TS2/7, BioLegend #328303), and anti-CD49b-PE-Cy7 (PIE6-C5, BioLegend #359313).

### 2.6. Expression of NKp44 Isoform Constructs

#### 2.6.1. Retroviral Transduction

Phoenix amphotrophic cells were transfected with NKp44 isoform expression plasmids using Lipofectamine™ 3000 (Invitrogen™, #L300001, Waltham, MA, USA). Retrovirus was collected after 24 h of culture at 32 °C, 5% CO_2_. 2B4 NFAT-GFP reporter cells were spinfected (2000 rpm, 26 °C) with virus containing supernatants in the presence of 1 μg/mL polybrene (Merk Millipore, #T$-1003-G, Darmstadt, Germany) for one hour. Then, 10 μg/mL puromycin (Tocris, #4089, Bristol, UK) was added after 48 h of culture in complete medium. After two weeks, NKp44^+^ cells were FACS sorted using anti-NKp44-AF647 (P44-8, BioLegend #325112) at the Melbourne Cytometry Platform ( Doherty Institute, Melbourne, VIC, Australia).

#### 2.6.2. Transient Transfection

A total of 10^5^ HEK 293T cells were seeded 48 h prior to transient transfection with DAP12-T2A/IRES-NKp44 plasmids using Lipofectamine™ 3000 (Invitrogen™, #L300001). After 24 h, NKp44 protein expression was determined at the cell surface by flow cytometry using anti-NKp44-AF647 (P44-8, BioLegend #315112) using an FACS Canto II (BD, Franklin Lakes, NJ, USA).

### 2.7. Flow Cytometry

#### 2.7.1. Surface Staining

Prior to use, all cells were washed twice in excess PBS and washed with PBS + 2% BCS in between antibody incubations. Propidium (PI) (Tocris, #5135) and Live/Dead Aqua (Invitrogen, #L34957) were used to determine cell viability in unfixed and fixed cells, respectively. Flow cytometry was performed on cells using an FACS Canto II or Fortessa (BD).

#### 2.7.2. Intracellular Staining

For antibody screening, control cells were labelled with Cell Trace Violet (CTV) (Invitrogen, #C34557A). As required, cells were fixed in 2% paraformaldehyde (PFA) (ProSciTech, #C004, Kirwan, QLD, Australia) for 10–15 min on ice. After sequential PBS washes, cells were permeabilised (FACS buffer + 0.1% saponin (Fisher Scientific, #S/0380/48, Waltham, MA, USA) before resuspension in primary anti-NKp44 isoform mAb diluted in permeabilization buffer at 4 °C. Unconjugated anti-NKp44 isoform mAbs were detected using Gαms-AF488 (Invitrogen, #A11029) diluted in permeabilization buffer for one hour on ice. For co-staining of surface markers, NK cells and PBMCs were blocked in permeabilization buffer containing 10% normal mouse serum (Abcam, #7486, Cambridge, UK) prior to staining with anti-CD3-BV421 (OKT3, BioLegend, #317344), anti-CD56-PE/Cyanine-7 (HCO56, BioLegend, #318318) and anti-NKp44-AF647 (P44-8, BioLegend, #325112) diluted in permeabilization buffer containing 10% normal mouse serum.

### 2.8. 2B4 NFAT-GFP Reporter Cell Assays

When required, flat-bottomed tissue-culture plates (Corning, #269620, NY, USA) were coated in purified hamster anti-mouse anti-CD3ε antibody (NA/LE, BD Pharmingen, #553057, Franklin Lakes, NJ, USA) or anti-NKp44 (P44-8, BioLegend, #325102) diluted in PBS overnight at 4 °C, or room temperature for 60 min. After PBS washing, 1–2 × 10^5^ 2B4 NFAT-GFP reporter cells were stimulated with or without increasing concentrations of recombinant human PDGF-DD (rhPDGF-DD) (R&D Systems, #SB1159, Minneapolis, MN, USA) for 16 h. For blocking experiments, cells were preincubated with 10 μg/mL anti-NKp44 (P44-8, BioLegend, #325102) mAb for at least 30 min at ambient temperature. The role of endocytosis was tested by stimulating NKp44-1 and NKp44-3 NFAT-GFP reporter cells in reduced serum conditions (5% BCS) with 250 ng/mL rhPDGF-DD either in the presence of 80 μM Dynasore (Tocris, #2897) or DMSO (Sigma, D4540-1L) as solvent-only control for 4 h prior to analysis by flow cytometry.

### 2.9. Confocal Microscopy

Chamber-slides (Lab-Tek, #154534, Waltham, MA, USA) were coated with Poly-L-lysine (Sigma, #P8920-100ML) for 10 min at ambient temperature. Control cell lines were washed in PBS, counted and fluorescently labelled with CTV according to the manufacturer’s protocol. After quenching excess dye, cells were mixed in equal numbers to achieve ~80% confluence upon adherence to the chamber-slides (20 min at 37 °C in PBS). After fixation in 2% PFA for 10–15 min at ambient temperature, cells were blocked in PBS + 2% BCS + 0.1% saponin for at least 30 min at room temperature. Cells were incubated with anti-NKp44 mAb diluted in PBS + 2% BCS + 0.1% saponin overnight at 4 °C. After sequential PBS washes, cells were incubated with secondary fluorescently conjugated goat anti-mouse AF-555 (Invitrogen, #A-21422) diluted in PBS + 2% BCS + 0.1% saponin for 1 h at 4 °C. In the absence of CTV, cell nuclei were stained with 5 μg/mL of Hoescht 33342 (Thermo Fisher Scientific, #62249, Waltham, MA, USA) for 10 min. After PBS washing, chamber-slides were mounted on coverslips using ProLong Gold Antifade (Invitrogen, #P10144) and stored at 4 °C. Images taken using a LSM780 (Zeiss, Baden-Württemberg, Germany) confocal microscope equipped with a 20× or 63× objective were processed in ImageJ (v1.54).

### 2.10. Statistical Analysis

Data presented as median with interquartile range (IQR) are representative of multiple biological experiments performed in replicates, as described in the figure legend. Statistical tests were selected based on the normality of the data and performed using GraphPad Prism (v9). Flow cytometry data were analysed in FlowJo (v10).

## 3. Results

### 3.1. Monoclonal Antibodies Targeting Unique Cytoplasmic Sequences Enable Specific Detection of NKp44 Isoforms in NK Cells

**Figure 1:**
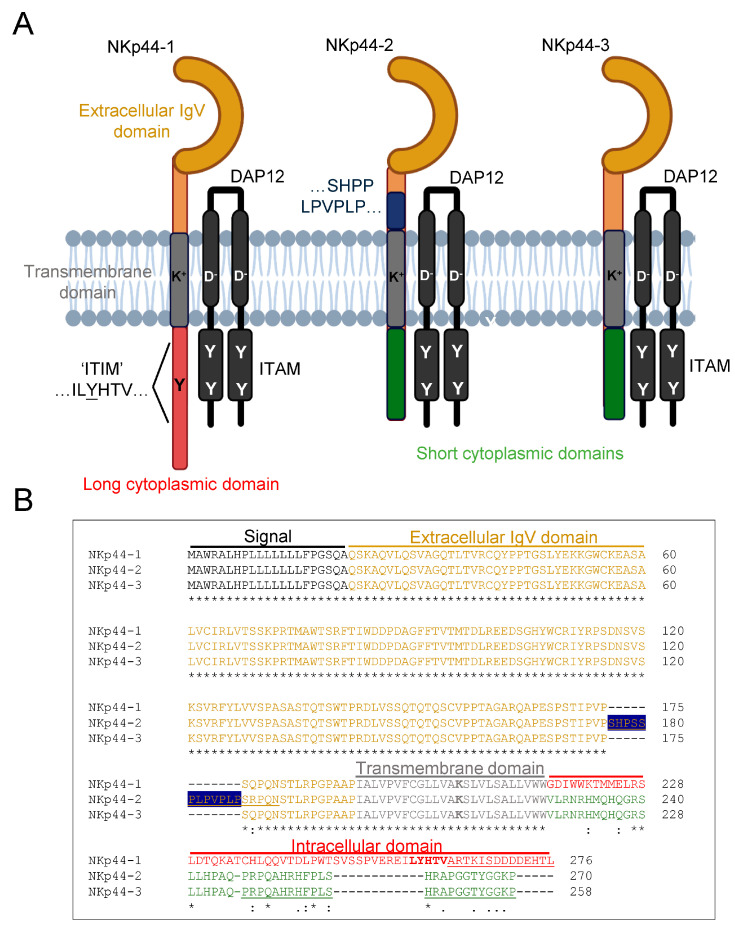
NKp44-1, but not NKp44-2 or NKp44-3, encodes a putative ITIM sequence in its cytoplasmic domain. (**A**) The long cytoplasmic tail of (left) NKp44-1 (red) encodes ILYTHV which resembles an ITIM (V/I/xYxxL/V). (middle) NKp44-2 and (right) NKp44-3 encode short cytoplasmic tails with no intrinsic signalling motifs. (**B**) Domain annotation of amino acid sequence alignment of NKp44-1 (NKp44-1: NP_004819), NKp44-2 (NP_001186438) and NKp44-3 (NP_001186439). Underlined sequences designate the linear peptides used for antibody generation: NKp44-1 (red), NKp44-2 (gold), NKp44-2/3 (green). Navy background highlights proline rich insertion unique to NKp44-2. Sequences were obtained from NCBI and aligned using the Uniprot Align tool https://www.uniprot.org/align (accessed on 29 August 2025).

Alternative splicing of *NCR2* has the potential to produce three NKp44 isoforms [13]. The transmembrane domain of all three NKp44 isoforms associates with the DAP12 adaptor protein, which is required for cell surface expression [17,22]. Although the extracellular V-type Ig domain is conserved between isoforms, in contrast to NKp44-2 and -3, NKp44-1 encodes a long cytoplasmic tail containing an ILYHTV sequence which conforms to a putative ITIM (Figure 1A). This cytoplasmic ITIM potentially endows NKp44-1 with the capacity for inhibitory signalling as well as activating signalling via DAP12 [17]. NKp44-1 expression has been linked with poor prognosis in cancer patients [21], and NK44-1 mRNA has been reported to be preferentially expressed in uterine uNK cells compared to the NKp44-2 and -3 mRNAs [20]. However, the lack of specific mAbs that can detect these proteins in cells and tissues has hindered our understanding of the functional roles of NKp44 receptor isoforms, particularly given that NKp44 ligands exhibit differential expression across various health and disease contexts [18].

**Figure 2:**
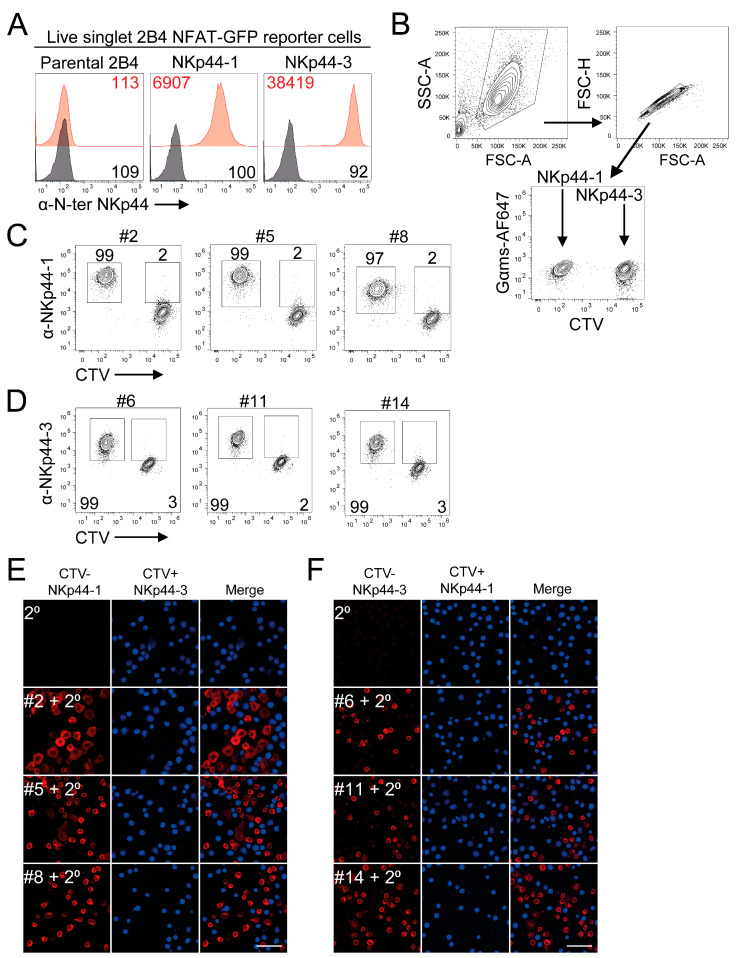
Identification of c-terminal targeting mAbs that specifically detect NKp44-1 and NKp44-3 by flow cytometry. (**A**) The stable expression of (red) NKp44-1 and NKp44-3 in polyclonal 2B4 NFAT-GFP reporter cells compared to parental 2B4 cells was determined by anti-NKp44-N-ter-AF647 (P44-8, BioLegend). (black) Unstained cells were used as controls. (**B**,**C**) Specific binding of anti-NKp44-1 mAbs #2, #5 and #8 to CTV- 2B4-NKp44-1 cells compared to CTV+ 2B4-NKp44-3 cells. Gates were set at approximately 1%+ NKp44-3 cells. (**D**) Specific binding of anti-NKp44-3 mAbs #6, #11 and #14 to CTV- 2B4-NKp44-3 cells compared to CTV+ 2B4-NKp44-1 cells. In both cases, N-terminal targeting of anti-NKp44 (P44-8, BioLegend) was used as a positive control. Anti-NKp44-C-ter mAbs were detected using a Gαms-AF647. Gates were set at approximately 2%+ control cells. Representative dot plots depict percent NKp44 isoform positive cells from three independent experiments. (**E**) Anti-NKp44-1 mAbs and (**F**) anti-NKp44-3 mAbs clones selectively bind CTV- 2B4-NKp44-1 and 2B4-NKp44-3 cells, respectively, compared to CTV-labelled control cells by confocal microscopy. Scale bar = 50 μm. Images are representative of at least five fields of view taken from two independent experiments.

To address this, we generated C-terminal-specific mAbs by immunizing mice with peptides derived from the unstructured cytoplasmic regions of NKp44-1 and NKp44-2/3 (Figure 1B, underlined). To evaluate the ability of mAbs to specifically recognise the different NKp44 isoforms, we engineered bicistronic DAP12-T2A-NKp44 constructs expressing either NKp44-1 or NKp44-3 in complex with DAP12, which is required for the cell surface expression of NKp44 [22,36]. The DAP12-T2A-NKp44-1 and DAP12-T2A-NKp44-3 constructs were expressed on the cell surface when transiently expressed in HEK 293T cells (Figure S1), and on the cell surface of 2B4 NFAT-GFP cells following stable selection in puromycin, respectively (Figure 2A) [33]. Next, the specificity of the anti-NKp44-1 mAbs was assessed by intracellular staining of a mixture of 2B4-NKp44-1 cells and cell-trace violet (CTV)-labelled 2B4-NKp44-3 cells, allowing the two isoform-expressing cell populations to be distinguished by flow cytometry (Figure 2B). Supernatants from anti-NKp44-1 mAb clones #2, #5 and #8 specifically stained 2B4-NKp44-1 cells (>99%) over CTV+ 2B4-NKp44-3 cells (Figure 2C). Using this approach, we also found that anti-NKp44-3 clones #6, #11 and #14 specifically stained 2B4-NKp44-3 cells (>99%) over CTV+ 2B4-NKp44-1 cells (Figure 2D). Peptides encompassing the unique extracellular sequence in NKp44-2 did not generate effective monoclonal antibodies. Given the importance of spatial relationships in immune cell function, we sought to determine whether these mAbs could be used by confocal microscopy. The anti-NKp44-1 and NKp44-3 mAbs specifically detected their respective isoform when mixed with CTV-labelled control 2B4 cells expressing the alternate isoform (Figure 2E,F). Overall, these results indicate that we have developed C-terminal mAbs that can detect the cytoplasmic tails of the NKp44-1 and NKp44-2/3 isoforms by flow cytometry and confocal microscopy.

**Figure 3:**
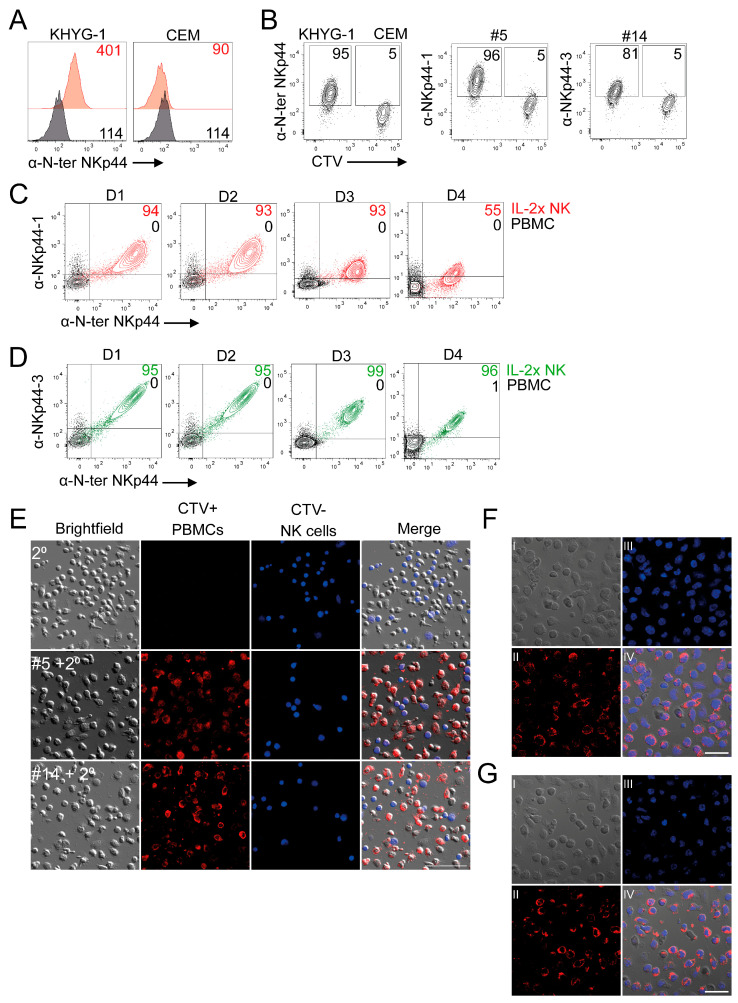
C-terminal targeting mAbs enable detection of NKp44-1 and NKp44-3 in human NK cells. (**A**) KHYG-1, but not CEM cells, display surface expression of NKp44. Surface NKp44 was detected using anti-NKp44-N-ter-AF647 (P44-8, BioLegend). (**B**) NKp44+ KHYG-1 NK cells were mixed with NKp44-deficient CTV-labelled CEM cells to assess the specificity of anti-NKp44-1 #5 mAb and anti-NKp44-3 #14 mAb. Gates were set at approximately 5%+ of control cells. Representative dot plots depict percent NKp44 isoform positive cells from two independent experiments. (**C**,**D**) Intracellular staining of IL-2 expanded NK cells compared with (black) resting PBMCs using (red) anti-NKp44-1 #5 and (green) anti-NKp44-3 #14. NKp44 isoform expression was correlated with total NKp44 by co-staining with anti-NKp44-N-ter-AF647. Dot plots depict percent double-positive IL-2 activated NK cells from four independent donors (D1–4) stained intracellularly with anti-NKp44 isoform mAbs. Anti-NKp44-1 and anti-NKp44-3 mAbs were detected with secondary Gαms-AF488 (Invitrogen). Quadrants were adjusted according to PBMCs stained with secondary antibody alone. (**E**) Confocal microscopy images of IL-2 activated NK cells mixed with CTV+ resting PBMCs stained with (top) Gαms-AF555 alone or in combination with either (middle) anti-NKp44-1 #5 or (bottom) anti-NKp44-3 #11. Visualisation of (**F**) NKp44-1 and (**G**) NKp44-3 in IL-2 activated NK cells using anti-NKp44-1 mAb and anti-NKp44-3 mAb. (**I**) brightfield, (**II**) anti-NKp44 isoform mAb + Gαms-AF555, (**III**) Hoescht 3342, (**IV**) merge. Scale bar = 50 μm. Images are representative of two independent experiments.

Although the relative expression of NKp44 isoforms has been previously been estimated using RT-PCR [20,21], the relative expression of NKp44-1 and NKp44-2/3 at the protein level in NK cell lines or primary cells is unknown. Therefore, we performed intracellular staining on mixtures of the NKp44^+^ KHYG-1 NK cell line and the NKp44-deficient human CEM T cell line labelled with CTV (Figure 3A) [34,35]. We found that anti-NKp44-1 clone #5 and anti-NKp44-2/3 clone #14 stained 96% and 81% of KHYG-1 cells, respectively, with minimal cross-reactivity in CEM cells (Figure 3B). These results suggest that these anti-NKp44 isoform mAbs specifically detect the endogenous expression of NKp44-1 and NKp44-2/3 in the human KHYG-1 NK cell line but not in the NKp44-deficient human CEM cell line.

As NKp44 is upregulated on NK cells by IL-2, we next sought to determine the expression of the NKp44 isoforms in ex vivo NK cells expanded in IL-2 and compared to resting peripheral blood mononuclear cells (PBMCs) that do not express NKp44 (Figure S2). We performed these experiments using IL-2 activated NK cells purified from four independent donors. To corroborate NKp44 isoform expression with that of total NKp44, we co-stained NK cells and PBMCs with a commercial anti-NKp44 mAb that recognises the N-terminus of NKp44 which is conserved across all NKp44 isoforms. Staining with the anti-NKp44-1 mAb #5 detected 94%, 93%, 93% and 53% double-positive IL-2 activated NK cells (Figure 3C), whereas the anti-NKp44-2/3 mAb #14 stained 95%, 95%, 99% and 96% double-positive IL-2 activated NK cells (Figure 3D) that were absent in the PBMC samples. Next, we assessed the ability of these mAbs to detect NKp44-1 and NKp44-2/3 isoforms in IL-2 expanded NK cells compared to CTV-labelled resting PBMCs by confocal microscopy. Anti-NKp44-1 #5 and anti-NKp44-2/3 #14 mAbs stained IL-2 expanded NK cells and displayed minimal cross-reactivity to CTV-labelled resting PBMCs (Figure 3E–G). Overall, using novel C-terminal targeting mAbs, we provide firsthand evidence that NKp44-1 and NKp44-2/-3 are expressed at the protein level in primary NK cells expanded in IL-2, but not in freshly isolated PBMCs, consistent with the known IL-2-inducible upregulation of NKp44 on human NK cells.

### 3.2. NKp44 Isoform Expression in NK Cell Subsets

**Figure 4:**
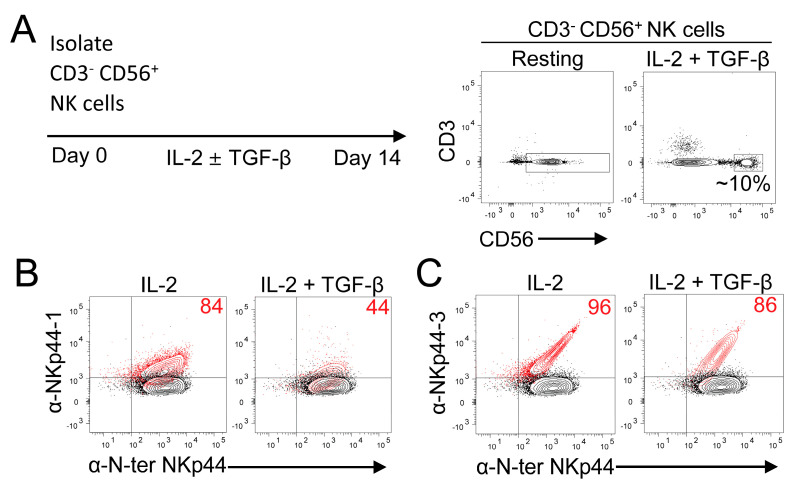
NK cell expression of NKp44-1 expression is not differentially upregulated by TGF-β in vitro. (**A**) Surface expression of CD3 and CD56 in freshly isolated NK cells, compared to “uterine-like” NK cells after 14 days of culture in TGF-β (10 ng/mL) and IL-2 (100 U/mL). Intracellular staining of (red) NK cells cultured in (left) IL-2 or (right) IL-2 + TGF-β with (**B**) anti-NKp44-1 #5 mAb and (**C**) anti-NKp44-3 #14 mAb co-stained with anti-N-ter-NKp44 (P44-8), compared to (black) IL-2 expanded NK cells stained with anti-N-ter-NKp44 (P44-8, BioLegend) alone. Anti-NKp44 isoform mAbs were detected with a secondary Gαms-AF488 (Invitrogen). Representative dot plots depict staining performed in duplicate from NK cells isolated from two independent donors.

Uterine NK cells (uNK) and peripheral blood NK cells (pNK) show distinct mRNA transcript profiles for the NKp44 isoforms; however, this has not been verified at the protein level [20]. To address this, we differentiated pNK cells into a “uterine-like” phenotype in vitro using TGF-β in combination with IL-2 [37]. In our hands, culture in a combination of IL-2 and TGF-β for 14 days induced a distinct population (~10%) of CD3^−^ CD56^superbright^ NK cells with a surface phenotype reminiscent of uNK, compared to resting NK (Figure 4A and Figure S3) [37,38,39]. Next, we performed intracellular staining using anti-NKp44-1 #5 and anti-NKp44-2/3 #14 mAbs. Compared to culture in IL-2 alone, the addition of TGF-β reduced the number of NKp44-1 expressing NK cells from 84% to 44% (Figure 4B), whereas NKp44-2/3 expression was only reduced by 10% (Figure 4C). The reduction in NKp44-1 protein expression in TGF-β differentiated uNK-like cells contrasts with previous reports of preferential expression of NKp44-1 mRNA transcripts in uNK [20]. These results suggest that differentiation of NK cells in TGF-β may downregulate NKp44-1 expression at the protein level.

### 3.3. Engagement of NKp44-1 with PDGF-DD Does Not Inhibit TCR Signalling

**Figure 5:**
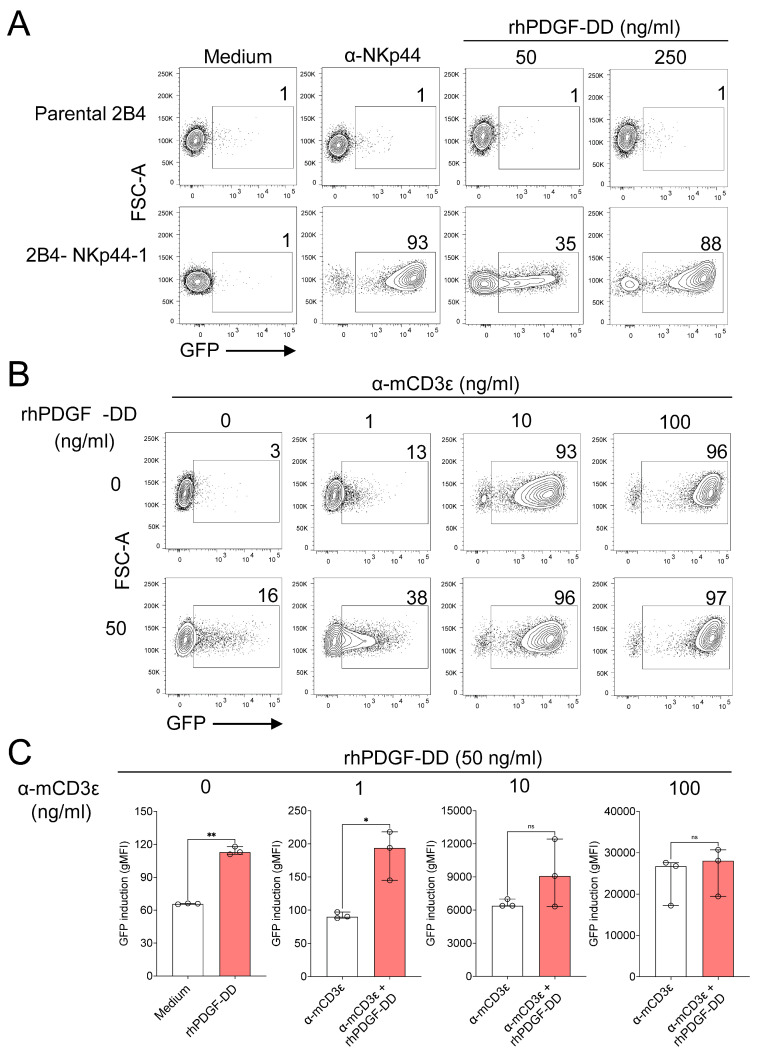
NKp44-1 does not inhibit activation of NFAT-GFP reporter cells by α-mCD3ε. (**A**) Representative dot plots depict GFP+ (%) (top) parental 2B4 cells and (bottom) 2B4-NKp44-1 cells either stimulated either with plate-bound anti-NKp44 mAb or 50 or 250 ng/mL rhPDGF-DD for 16 h. (**B**) GFP+ (%) and (**C**) GFP signal intensity (gMFI) in 2B4-NKp44-1 cells stimulated with 50 ng/mL rhPDGF-DD in the presence of 0, 1, 10, or 100 ng/mL plate-bound anti-mCD3ε antibody for 16 h. Representative dot plots and median with IQR gMFI data are representative of three independent experiments performed in triplicate. Statistical significance was determined using Welch’s *t* tests. * *p* < 0.05, ** *p* < 0.01.

Having provided evidence that the NKp44-1 isoform is expressed at the protein level in NK cells, we next sought to understand how NKp44-1 signals in the context of PDGF-DD compared to the NKp44-3 isoform, which lacks the cytoplasmic tyrosine-based signalling motif (Figure 1B). Whilst PDGF-DD triggers NK cell secretion of cytokines, degranulation and cytotoxicity via NKp44 ([22]; Sedgwick et al. under submission), NKp44-1 has potential to function as an immune checkpoint [40,41,42]. Whilst a capacity for dual signalling could potentially position NKp44-1 as a critical regulator of NK cell activity in contexts such as tumour immune evasion and maternal-fetal tolerance, whether NKp44-1 is inhibitory upon binding to PDGF-DD remains unclear. To investigate this, we examined whether PDGF-DD can activate NKp44-1 expressing NFAT-GFP reporter cells. Stimulation with 50 or 250 ng/mL of rhPDGF-DD for 16 h triggered GFP expression in 2B4-NKp44-1 cells, whereas NKp44-deficient parental 2B4 cells showed no GFP response (Figure 5A). These results indicate that PDGF-DD stimulation is sufficient to drive NKp44-1-dependent NFAT activation.

Although these data suggested that NKp44-1 delivers activating signals when stimulated with PDGF-DD, we next set out to determine whether NKp44-1 might inhibit signalling when either stimulated with rhPDGF-DD or co-ligated to another activating receptor. To address this, we investigated the effect of simultaneously triggering NKp44-1 and the murine T cell receptor TCR (mTCR), which utilises ITAM signalling and is endogenously expressed on 2B4 NFAT-GFP reporter cells (Figure S4A) [43]. Co-ligation of the mTCR to NKp44-1 using plate-bound anti-mCD3ε and anti-NKp44 mAbs increased the number of GFP-producing cells as well as GFP signal strength, indicating that NKp44-1 co-stimulated mTCR signalling (Figure S4B–D). Stimulation with 50 ng/mL (Figure 5B,C) and 250 ng/mL (Figure S4E,F) of rhPDGF-DD also triggered an increase in GFP production in NKp44-1-expressing cells co-stimulated with 1, 10 and 100 ng/mL of plate-bound anti-mCD3ε antibody. Collectively, these data indicate greater NFAT activation upon co-engagement of NKp44-1 and the mTCR with either anti-NKp44 mAb or PDGF-DD as NKp44-1 ligand, and are consistent with NKp44-1 functioning as an activating, rather than inhibitory, receptor.

### 3.4. NKp44-1 and NKp44-3 Trigger NFAT Activation When Stimulated with PDGF-DD

**Figure 6:**
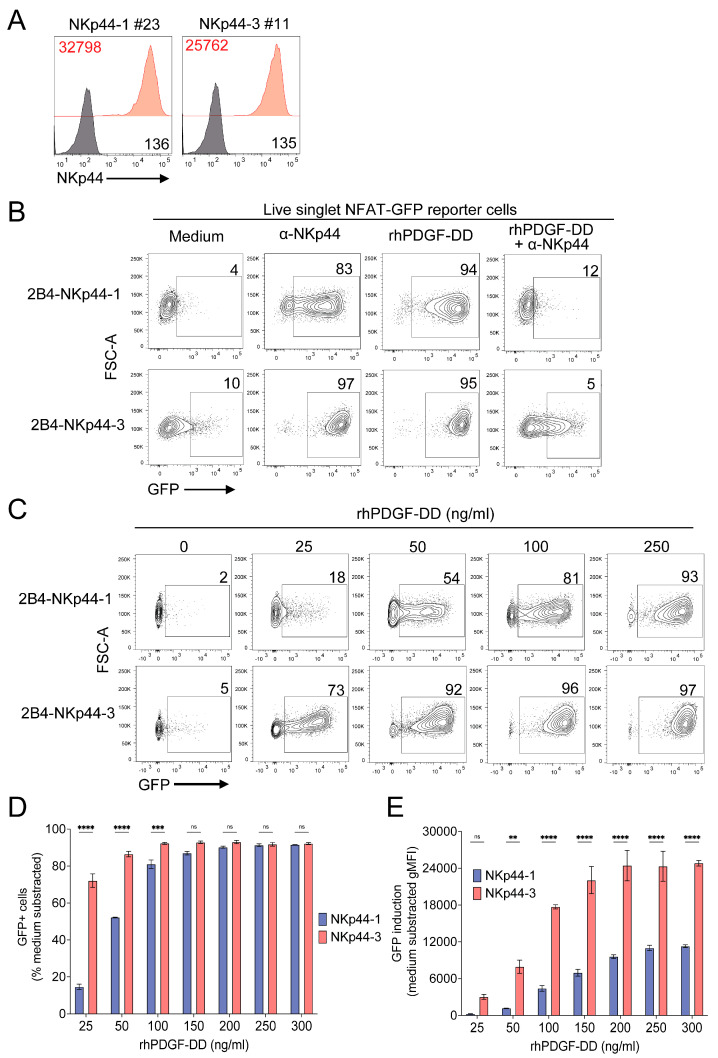
NKp44-3 triggers greater cellular activation than NKp44-1 in response to PDGF-DD. (**A**) NKp44-1 and NKp44-3 surface expression in single cell 2B4 NFAT-GFP reporter cells Representative histograms depict mean intensity fluorescence of anti-NKp44 antibody (red) compared to unstained (black) cells. from two experiments. (**B**) Representative dot plots depict GFP production in (top) NKp44-1 #23 and (bottom) NKp44-3 #11 cells at (left–right) rest, stimulated with plate bound anti-NKp44 antibody (10 μg/mL) or rhPDGF-DD (250 ng/mL) in the absence or presence of blocking anti-NKp44 antibody. (**C**) Representative dot plots of GFP+ cells (%) after stimulation of 2B4-NKp44-1 and 2B4-NKp44-3 clones with 25, 50, 100, 250 ng/mL rhPDGF-DD. (**D**) GFP+ (%) cells and (**E**) mean intensity fluorescence of GFP in (mauve) NKp44-1 #23 and (pink) NKp44-3 #11 cells after stimulation with 0, 25, 50, 100, 150, 200, 250 and 300 ng/mL rhPDGF-DD for 16 h. Representative plots depict median with range from two independent experiments performed in triplicate. Statistical significance was calculated using 2-way ANOVA with Šidák’s multiple comparisons test. ** *p* < 0.01, *** *p* < 0.001, **** *p* < 0.0001.

Although our observations indicate that PDGF-DD binding to NKp44-1 induced cellular activation, we remained intrigued by the possibility that the ITIM might differentially regulate NKp44-1 signalling compared to NKp44-2/3. To address this, we compared the effect of PDGF-DD stimulation through NKp44-1 to the archetypal activating NKp44-3 isoform expressed in 2B4 NFAT-GFP reporter cells (Figure 1A). Given the higher surface expression of NKp44-3 compared to NKp44-1 when expressed in polyclonal stable lines (Figure 2A), it was unsurprising that NKp44-3 induced a significantly stronger NFAT-GFP activation when stimulated with rhPDGF-DD (Figure S5). To control for the disparity in surface expression in the polyclonal cell lines, we generated single cell clones that stably expressed comparable levels of NKp44-1 (NKp44 gMFI: 36,429) and NKp44-3 (NKp44 gMFI: 26,597) (Figure 6A).

Similarly to polyclonal cells, single cell clones expressing NKp44-1 and NKp44-3 retained NFAT-GFP activation following stimulation with either plate-bound anti-NKp44 mAb or 250 ng/mL rhPDGF-DD (Figure 6B). Although comparable frequencies of GFP+ cells were observed following stimulation with concentrations of rhPDGF-DD > 100 ng/mL (Figure 6C,D), the signal strength—reflected by the mean fluorescent intensity of GFP expression—was consistently lower for NKp44-1 than for NKp44-3 across all PDGF-DD concentrations (Figure 6E). Together, these findings demonstrate that both NKp44-1 and NKp44-3 can trigger NFAT activation in response to PDGF-DD, albeit with consistently weaker signalling mediated through NKp44-1 compared to NKp44-3.

### 3.5. The Cytoplasmic Y238 of NKp44-1 Is Dispensable for PDGF-DD Triggering of Activating Signalling in 2B4 NFAT-GFP Reporter Cells

**Figure 7:**
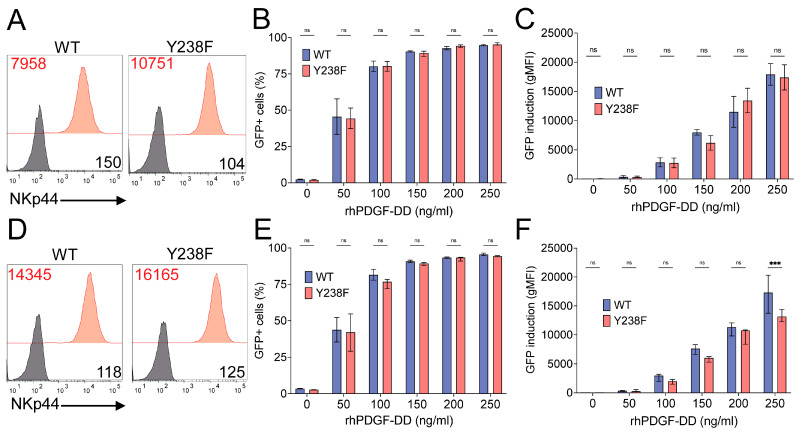
Y238 is dispensable for NKp44-1 triggering of cellular activation in response to PDGF-DD. (**A**) NKp44 surface expression (red) in NKp44-1 #13 and NKp44-1 Y238F #16 cells compared to unstained (black) cells. (**B**) GFP production (% GFP+ cells) and (**C**) intensity of GFP signal in NKp44-1 #13 (mauve) compared to NKp44-1 Y238F #16 (red) cells treated with increasing concentrations of 0, 50, 100, 150, 200 and 250 ng/mL rhPDGF-DD. (**D**) NKp44 surface expression (red) in NKp44-1 #16 and NKp44-1 Y238F #15 cells compared to unstained (black) cells. (**E**) GFP production (% GFP+ cells) and (**F**) intensity of GFP signal in NKp44-1 #13 (black) compared to NKp44-1 Y238F #16 (red) cells treated with 0, 50, 100, 150, 200 and 250 ng/mL rhPDGF-DD for 16 h. NKp44 expression data and scatter plots that depict median with IQR are representative of three independent experiments performed in triplicate. Statistical significance was calculated using Šidák’s multiple comparisons test. *** *p* < 0.001.

Given previous reports that the cytoplasmic Y238 of NKp44-1 can mediate inhibitory signalling [40], we next investigated how this residue might influence NKp44-1 activation in response to PDGF-DD stimulation. We generated 2B4 NFAT-GFP cells that stably express an NKp44-1 construct in which the cytoplasmic tyrosine at position 238 was replaced with phenylalanine (Y238F), a mutation that prevents tyrosine phosphorylation at this site and blocks the recruitment of SHP-1, SHP-2, or SHIP that can mediate inhibitory signalling [17]. To account for differences in surface expression that may influence the strength of downstream signalling, we identified two pairs of clones that expressed similar surface levels of wildtype (wt) NKp44-1 and NKp44-1 Y238F proteins (Figure 7A). Stimulation with increasing PDGF-DD concentrations induced comparable numbers of GFP+ cells and GFP signal intensity from wt NKp44-1 clone #13 (NKp44, gMFI = 7958) and NKp44-1 Y238F clone #16 (NKp44, gMFI = 10,751) (Figure 7B,C). A similar pattern was also observed for another set of clones with comparable surface expression of wtNKp44-1 (clone #16, gMFI = 14,345) or NKp44-1 Y238F (clone #15, gMFI = 16,165) (Figure 7D–F). Overall, these results indicated that the cytoplasmic Y238 is dispensable for transducing activating signals downstream of PDGF-DD binding to NKp44-1 in 2B4 NFAT-GFP reporter cells.

### 3.6. Y238 Regulates the Cell Surface Expression of NKp44-1

**Figure 8:**
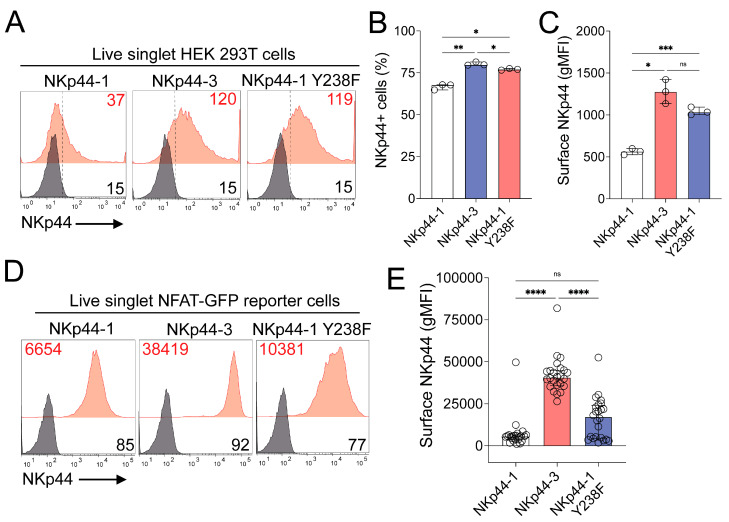
Y238 regulates NKp44-1 surface expression in HEK 293T and NFAT-GFP reporter cells. (**A**) Representative surface expression of NKp44 (red) in HEK 293T cells transiently transfected to express NKp44-1, NKp44-3 or NKp44-1 Y238F, compared to unstained (black) cells. Quantification of (**B**) NKp44+ (%) cells and (**C**) NKp44 surface expression (gMFI) in transiently transfected HEK 293T cells. Representative median with IQR NKp44 expression data are representative of duplicate staining of HEK 293T cells from three independent transfections. Statistical significance was calculated using Dunnett’s T3 multiple comparisons test. * *p* < 0.05, ** *p* < 0.01, *** *p* < 0.001. (**D**) NKp44 surface expression (red) in polyclonal NFAT-GFP reporter cells expressing NKp44-1, NKp44-3 and NKp44-1 Y238F, compared to unstained (black) cells. Representative histogram depicts gMFI from two independent experiments. (**E**) Median with IQR of mean intensity fluorescence of NKp44 surface expression in 24 single cell clones stably expressing NKp44-1, NKp44-3 or NKp44-1 Y238F. Each point represents a single clone. Statistical significance was calculated using Dunnett’s T3 multiple comparisons tests. **** *p* < 0.0001.

We searched The Eukaryotic Linear Motif Resource for Functional Sites in Proteins (ELM) for functional motifs in the NKp44-1 (Table S1) and NKp44-3 (Table S2) cytoplasmic tails that may provide insights into their regulatory or signalling functions [44]. Intriguingly, the Y238-based motif “YHTV” in NKp44-1 was also identified as a potential Yxxϕ endocytosis motif, where ϕ denotes any amino acid with a bulky hydrophobic side chain [15]. We hypothesised that Y238 may mediate greater internalisation, which may reduce the surface expression of NKp44-1. In support of this, NKp44-1 displayed significantly lower cell surface expression than NKp44-3 when transiently transfected into HEK 293T cells (Figure 8A–C), and reduced NKp44-1 surface expression was also observed in polyclonal 2B4 NFAT-GFP reporter cells stably expressing NKp44-1 (Figure 8D) and in most clones that stably expressed NKp44-1 generated in this system compared to NKp44-3 (Figure 8E). Additional support for YHTV as a Yxxϕ endocytic motif was observed when comparing surface expression of NKp44-1 Y238F mutant with wtNKp44-1. NKp44-1-Y238F consistently displayed higher levels in transiently transfected HEK 293T cells (Figure 8A–C) and at the surface of polyclonal and monoclonal 2B4 NFAT GFP reporter cells (Figure 8D,E). Altogether, these results suggest that the cytoplasmic Y238 residue may mediate NKp44-1 cell surface expression as an endocytic cell trafficking rather than inhibitory signalling motif.

### 3.7. Inhibition of Endocytosis Diminishes NKp44 Signalling in Response to PDGF-DD

**Figure 9:**
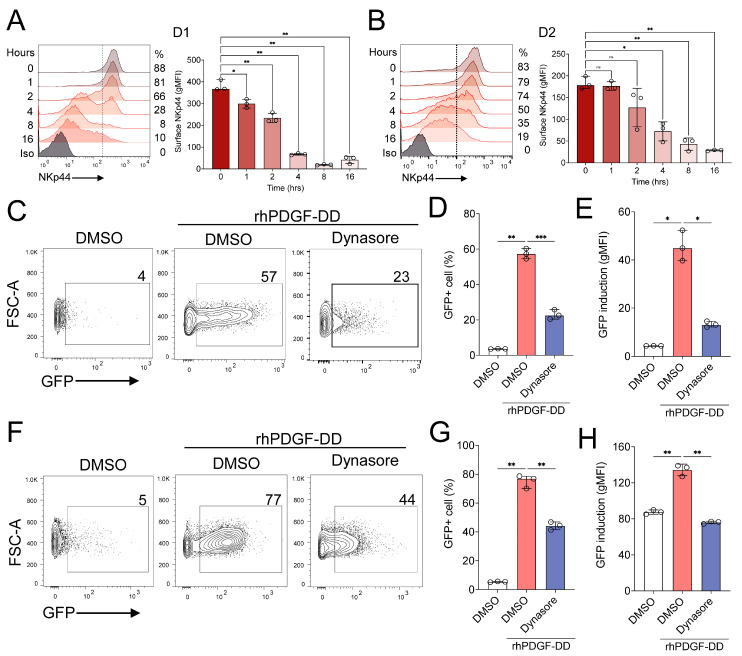
PDGF-DD-NKp44 signalling is sensitive to endocytosis inhibition. (**A**,**B**) NKp44+ cells (%) and surface expression of NKp44 (gMFI) in IL-2 activated NK cells from two independent donors (D1, D2) after stimulation with rhPDGF-DD (250 ng/mL) for 0, 1, 2, 4, 8, 16 h as determined by anti-NKp44-AF647 mAb (P44-8, BioLegend) by flow cytometry. The percentage of NKp44^+^ cells was determined relative to the 0 h timepoint. Representative histograms and bar graphs depict median with IQR from three independent experiments performed in triplicate from two independent donors (D1, D2). Statistical significance was tested using Dunnett’s T3 multiple comparison test. * *p* < 0.05, ** *p* < 0.01. Effect of Dynasore treatment on NFAT-GFP activation in (**C**–**E**) 2B4-NKp44-1 and (**F**–**H**) 2B4-NKp44-3 reporter cells. Representative dot plots depict GFP+ cells (%) and representative column graphs depict median with IQR data from three independent experiments performed in triplicate. Statistical significance was calculated using Dunn’s multiple comparison test. * *p* < 0.05, ** *p* < 0.01, *** *p* < 0.001.

The possibility that Y238 regulates NKp44-1 surface expression prompted us to investigate the role of endocytosis in NKp44-1 signalling. Initially, we observed that PDGF-DD stimulation induces a progressive loss of NKp44 from the NK cell surface over time (Figure 9A,B). This raised the question of whether endocytosis is required for the propagation of PDGF-DD-NKp44 signalling. To assess this, we measured the activation of NKp44-1 and NKp44-3 NFAT-GFP reporter cells in response to PDGF-DD in the presence or absence of the dynamin-2 inhibitor dynasore [45]. To mitigate the effects of DMSO on cell viability and receptor dynamics, we optimized the dynamin inhibition assay to measure activation of 2B4-NKp44 reporter cells after four hours of incubation with 250 ng/mL of rhPDGF-DD. We observed that dynasore significantly reduced the signal strength and number of GFP producing 2B4-NKp44-1 cells compared to cells treated with control DMSO solvent that were stimulated with rhPDGF-DD (Figure 9C–E). Interestingly, dynasore also significantly weakened the GFP signal intensity and reduced the number of GFP+ 2B4-NKp44-3 cells (Figure 9F–H). Altogether, these results suggest that optimal signalling from either NKp44-1 or NKp44-3 may rely on receptor-mediated endocytosis, which may explain the loss of NKp44 from the NK cell surface upon stimulation with soluble PDGF-DD.

## 4. Discussion

Understanding how receptor–ligand interactions shape NK cell activity across diverse physiological and pathological settings is crucial for developing strategies that effectively leverage NK cells for immunotherapy [46]. Although PDGF-DD is known to engage the NKp44 immunoreceptor and drive NK cell activation, the specific roles of the individual NKp44 isoforms in directing downstream signalling and cell trafficking events remain poorly defined [22]. Our study provides a revised and integrated model of NKp44 isoform biology that reshapes how NKp44 signalling is understood in the context of its secreted ligand, PDGF-DD. A central advance of this work is the first direct demonstration that the NKp44-1 and NKp44-2/3 proteins are expressed in human primary NK cells, enabled by the isoform-specific C-terminal mAbs that we have generated. Despite two decades of assumptions based on mRNA expression profiles, until now it has remained unproven whether the NKp44 splice variants were translated into distinct protein isoforms. By validating unique C-terminal epitopes, establishing isoform specificity in stably transfected cells, and applying these mAbs to primary IL-2–activated NK cells and human NK cell lines, we show unequivocally that both NKp44-1 and NKp44-2/3 are present at the protein level in primary IL-2 expanded NK cells but not resting NK or other PBMC populations. This foundational result resolves a long-standing uncertainty and provides essential tools for studying isoform-specific signalling in human cells and tissues. 

As we can now distinguish NKp44 isoforms at the protein level, we were able to test how cytokines, such as TGF-β that are critical for the development of tissue-resident NK cell populations, such as uNK, may potentially shape NKp44 isoform expression. In uterine-like NK cells differentiated with TGF-β, we found selective downregulation of NKp44-1 compared to NKp44-3, contradicting earlier transcript-only studies that suggested preferential NKp44-1 enrichment in uterine NK cells [20]. This highlights the critical importance of protein-level isoform detection and suggests that local signals such as TGF-β selectively tune NKp44 isoform balance, potentially affecting NK responsiveness to PDGF-DD in tissues such as the decidua or PDGFD-expressing tumours.

With protein-level detection established, our functional analyses reveal that NKp44-1 is not an inhibitory receptor for PDGF-DD, despite possessing a cytoplasmic ITIM-like motif. Instead, NKp44-1 activates NFAT signalling. This overturns the long-standing assumption that NKp44-1 is intrinsically inhibitory and demonstrates that, when engaged by physiological ligand, NKp44-1 functions as an activating receptor—though it consistently elicits weaker signalling than NKp44-3 even at matched surface expression levels, revealing intrinsic isoform-specific tuning downstream of a shared ligand (e.g., divergent associations with signalling partners) that might govern intrinsic differences between NKp44-1 and NKp44-3 signalling. Here, we addressed the specific question of whether Y238 mediates an inhibitory contribution to NKp44-1 signalling by employing a Y>F substitution in the ITIM motif, a well-established strategy that prevents tyrosine phosphorylation and thereby abrogates ITIM-dependent recruitment of SH2-domain phosphatases [47]. Our data show that the cytoplasmic Y238 of NKp44-1 is dispensable for activation, indicating that the ITIM-like sequence does not recruit inhibitory phosphatases after PDGF-DD engagement. Instead, Y238 behaves as a YxxΦ-type endocytic motif, because substitution of the Y238 for phenylalanine increases NKp44-1 surface abundance. Although variants of YxxΦ motifs have been described in which additional upstream hydrophobic residues contribute to AP-2 binding (e.g., the “three-pin plug” model proposed for P-selectin), NKp44-1 lacks such a residue at the Y–3 position, suggesting that Y238 itself represents the dominant determinant governing receptor internalization [48]. These findings unify prior observations that the Y238F mutation increases NKp44-1 surface expression on NK cells and is dispensable for NK cell activation [17], thus establishing a model in which Y238 tunes NKp44-1 surface residency and availability for ligand engagement.

Our data show that PDGF-DD stimulation induces NKp44 internalisation in primary NK cells and that dynamin inhibition dampens NFAT activation for both NKp44-1 and NKp44-3. In addition to this finding, the Y238 dependence of surface expression reveals a broader principle: NKp44 signalling is coupled to receptor endocytosis when engaged by PDGF-DD, and internalization may contribute to signal propagation, potentially from within early endosomal compartments. This aligns NKp44 with other hybrid ITAM/ITIM or endosome-signalling receptors such as KIR2DL4 and SIGLEC-15 [49,50], supporting the concept that NKp44—particularly NKp44-1—may operate from within intracellular signalling microenvironments [51,52]. In this regard, NKp44 recognition of PDGF-DD has been shown to synergize with signalling from Toll-like receptor 9 (TLR9)—which senses CpG-ODN within the endosomal compartments—to enhance pDC secretion of IFN-α, TNF and IL-6 [53]. Thus, it is tempting to speculate that NKp44-1 might preferentially signal from within endosomal compartments to allow NKp44-1 to cooperate with other endo-lysosomal receptors, such as TLR9 [54], potentially coordinating immune responses across diverse NKp44-expressing cell subsets in physiological settings where PDGF-D is expressed.

## 5. Conclusions

By providing the first protein-specific detection tools and demonstrating isoform expression in primary cells, we have established a framework for studying NKp44 isoforms across NK cells, innate lymphoid cells of group 1 (ILC1) [22,55] and group 3 (ILC3) [56], multiple T cell subsets [57,58,59,60], and pDCs [36]. This will enable future investigations into how NKp44 isoform balance, trafficking, and endosomal signalling shape immunity across cancer, pregnancy, and inflammation. Moving forward, biochemical confirmation of adaptor interactions (e.g., AP-2 recruitment), live-cell imaging of NKp44 trafficking, and in-tissue mapping of isoform expression will be essential to refining the mechanistic model we propose. In summary, our study not only redefines how NKp44-1 and NKp44-3 signal but also closes a fundamental knowledge gap by demonstrating that the NKp44 isoforms are, indeed, expressed as proteins in primary human NK cells, providing tools and a conceptual foundation to enable future research on the role of NKp44 and PDGF-D.

## Data Availability

Original data will be made available upon reasonable request directed to the corresponding author.

## Supplementary Materials

The following supporting information can be downloaded at: https://www.mdpi.com/article/10.3390/cancers18071099/s1, Figure S1: Surface expression of DAP12-T2A-NKp44 constructs in HEK 293T cells; Figure S2: NK cell expression of NKp44 is inducible upon IL-2 activation; Figure S3: Expression of uterine NK cell markers in NK cells cultured in IL-2 with or without TGF-β; Figure S4: Stimulation of NKp44-1 enhances NFAT-GFP activation in response to mTCR agonism; Figure S5: Differential activation response to free PDGF-D in polyclonal NKp44-1 and NKp44-3 NFAT-GFP reporter cells; Table S1: The predicted functional motifs embedded in the cytoplasmic domain of NKp44-1; Table S2: The predicted functional motifs embedded in the cytoplasmic domain of NKp44-3.

## References

[1] Lanier L.L. (2008). Up on the Tightrope: Natural Killer Cell Activation and Inhibition. Nat. Immunol..

[2] Parham P. (2005). MHC Class I Molecules and Kirs in Human History, Health and Survival. Nat. Rev. Immunol..

[3] Ljunggren H.-G., Kärre K. (1990). In Search of the ‘Missing Self’: MHC Molecules and NK Cell Recognition. Immunol. Today.

[4] Bauer S., Groh V., Wu J., Steinle A., Phillips J.H., Lanier L.L., Spies T. (1999). Activation of NK Cells and T Cells by NKG2D, a Receptor for Stress-Inducible MICA. Science.

[5] Cosman D., Müllberg J., Sutherland C.L., Chin W., Armitage R., Fanslow W., Kubin M., Chalupny N.J. (2001). ULBPs, Novel MHC Class I–Related Molecules, Bind to CMV Glycoprotein UL16 and Stimulate NK Cytotoxicity through the NKG2D Receptor. Immunity.

[6] Chalupny N.J., Sutherland C.L., Lawrence W.A., Rein-Weston A., Cosman D. (2003). ULBP4 Is a Novel Ligand for Human NKG2D. Biochem. Biophys. Res. Commun..

[7] Bacon L., Eagle R.A., Meyer M., Easom N., Young N.T., Trowsdale J. (2004). Two Human ULBP/RAET1 Molecules with Transmembrane Regions Are Ligands for NKG2D. J. Immunol..

[8] Alexander David B., Marco C. (2019). Exploiting NK Cell Surveillance Pathways for Cancer Therapy. Cancers.

[9] Ma S., Yu J., Caligiuri M.A. (2025). Natural Killer Cell–Based Immunotherapy for Cancer. J. Immunol..

[10] Vitale M., Bottino C., Sivori S., Sanseverino L., Castriconi R., Marcenaro E., Augugliaro R., Moretta L., Moretta A. (1998). NKp44, a Novel Triggering Surface Molecule Specifically Expressed by Activated Natural Killer Cells, Is Involved in Non–Major Histocompatibility Complex–Restricted Tumor Cell Lysis. J. Exp. Med..

[11] Cantoni C., Bottino C., Vitale M., Pessino A., Augugliaro R., Malaspina A., Parolini S., Moretta L., Moretta A., Biassoni R. (1999). NKp44, a Triggering Receptor Involved in Tumor Cell Lysis by Activated Human Natural Killer Cells, Is a Novel Member of the Immunoglobulin Superfamily. J. Exp. Med..

[12] Lanier L.L., Corliss B.C., Wu J., Leong C., Phillips J.H. (1998). Immunoreceptor DAP12 Bearing a Tyrosine-Based Activation Motif Is Involved in Activating NK Cells. Nature.

[13] Allcock R.J.N., Barrow A.D., Forbes S., Beck S., Trowsdale J. (2003). The Human TREM Gene Cluster at 6p21.1 Encodes Both Activating and Inhibitory Single IgV Domain Receptors and Includes NKp44. Eur. J. Immunol..

[14] Ravetch J.V., Lanier L.L. (2000). Immune Inhibitory Receptors. Science.

[15] Bonifacino J.S., Traub L.M. (2003). Signals for Sorting of Transmembrane Proteins to Endosomes and Lysosomes. Annu. Rev. Biochem..

[16] Yusa S.-I., Campbell K.S. (2003). Src Homology Region 2-Containing Protein Tyrosine Phosphatase-2 (SHP-2) Can Play a Direct Role in the Inhibitory Function of Killer Cell Ig-like Receptors in Human NK Cells. J. Immunol..

[17] Campbell K.S., Yusa S., Kikuchi-Maki A., Catina T.L. (2004). NKp44 Triggers NK Cell Activation through DAP12 Association That Is Not Influenced by a Putative Cytoplasmic Inhibitory Sequence. J. Immunol..

[18] Barrow A.D., Colonna M. (2019). The Natural Cytotoxicity Receptors in Health and Disease. Front. Immunol..

[19] Niehrs A., Garcia-Beltran W.F., Norman P.J., Watson G.M., Holzemer A., Chapel A., Richert L. (2019). A Subset of HLA-DP Molecules Serve as Ligands for the Natural Cytotoxicity Receptor NKp44. Nat. Immunol..

[20] Siewiera J., Gouilly J., Hocine H.-R., Cartron G., Levy C., Al-Daccak R., Jabrane-Ferrat N. (2015). Natural Cytotoxicity Receptor Splice Variants Orchestrate the Distinct Functions of Human Natural Killer Cell Subtypes. Nat. Commun..

[21] Shemesh A., Brusilovsky M., Hadad U., Teltsh O., Edri A., Rubin E., Campbell K.S., Rosental B., Porgador A. (2016). Survival in Acute Myeloid Leukemia Is Associated with NKp44 Splice Variants. Oncotarget.

[22] Barrow A.D., Edeling M.A., Trifonov V., Luo J., Goyal P., Bohl B., Bando J.K., Kim A.H., Walker J., Andahazy M. (2018). Natural Killer Cells Control Tumor Growth by Sensing a Growth Factor. Cell.

[23] Yang L., Ren S., Lou L., He J., Huang Q., Wu X., Zhao R. (2025). A Bioinformatics Analysis and Experimental Validation of PDGFD as a Promising Diagnostic Biomarker for Acute Myeloid Leukemia. Sci. Rep..

[24] Jin K., Qiu S., Jin D., Zhou X., Zheng X., Li J., Liao X., Yang L., Wei Q. (2021). Development of Prognostic Signature Based on Immune-Related Genes in Muscle-Invasive Bladder Cancer: Bioinformatics Analysis of TCGA Database. Aging.

[25] Sun Y., Sedgwick A.J., Khan M.A.-A.-K., Palarasah Y., Mangiola S., Barrow A.D. (2021). A Transcriptional Signature of IL-2 Expanded Natural Killer Cells Predicts More Favorable Prognosis in Bladder Cancer. Front. Immunol..

[26] Xiaoqin Z., Zhouqi L., Huan P., Xinyi F., Bin S., Jiming W., Shihui L., Bangwei Z., Jing J., Yi H. (2024). Development of a Prognostic Signature for Immune-Associated Genes in Bladder Cancer and Exploring Potential Drug Findings. Int. Urol. Nephrol..

[27] Hu J., Wang L., Li L., Wang Y., Bi J. (2022). A Novel Focal Adhesion-Related Risk Model Predicts Prognosis of Bladder Cancer—A Bioinformatic Study Based on TCGA and GEO Database. BMC Cancer.

[28] Sun Y., Sedgwick A.J., Palarasah Y., Mangiola S., Barrow A.D. (2021). A Transcriptional Signature of PDGF-DD Activated Natural Killer Cells Predicts More Favorable Prognosis in Low-Grade Glioma. Front. Immunol..

[29] Li Y., Zhao Y., Shi M., Ma X., Jia M., Shen Z., Liu X., Li Y., Zhao L. (2025). PDGF-D Promotes Epithelial-Mesenchymal Transition of Glioma Cells Through the NF-κB/NOTCH1 Pathway. Cancer Med..

[30] Köhler G., Milstein C. (1975). Continuous Cultures of Fused Cells Secreting Antibody of Predefined Specificity. Nature.

[31] Pear W.S., Nolan G.P., Scott M.L., Baltimore D. (1993). Production of High-Titer Helper-Free Retroviruses by Transient Transfection. Proc. Natl. Acad. Sci. USA.

[32] Graham F.L., Smiley J., Russell W.C., Nairn R. (1977). Characteristics of a Human Cell Line Transformed by DNA from Human Adenovirus Type 5. J. Gen. Virol..

[33] Arase H., Mocarski E.S., Campbell A.E., Hill A.B., Lanier L.L. (2002). Direct Recognition of Cytomegalovirus by Activating and Inhibitory NK Cell Receptors. Science.

[34] Suck G., Branch D.R., Smyth M.J., Miller R.G., Vergidis J., Fahim S., Keating A. (2005). KHYG-1, a Model for the Study of Enhanced Natural Killer Cell Cytotoxicity. Exp. Hematol..

[35] Foley G.E., Lazarus H., Farber S., Uzman B.G., Boone B.A., Mccarthy R.E. (1965). Continuous Culture of Human Lymphoblasts from Peripheral Blood of a Child with Acute Leukemia. Cancer.

[36] Fuchs A., Cella M., Kondo T., Colonna M. (2005). Paradoxic Inhibition of Human Natural Interferon-Producing Cells by the Activating Receptor NKp44. Blood.

[37] Keskin D.B., Allan D.S.J., Rybalov B., Andzelm M.M., Stern J.N.H., Kopcow H.D., Koopman L.A., Strominger J.L. (2007). TGFbeta Promotes Conversion of CD16+ Peripheral Blood NK Cells into CD16- NK Cells with Similarities to Decidual NK Cells. Proc. Natl. Acad. Sci. USA.

[38] Du X., Zhu H., Jiao D., Nian Z., Zhang J., Zhou Y., Zheng X., Tong X., Wei H., Fu B. (2022). Human-Induced CD49a+ NK Cells Promote Fetal Growth. Front. Immunol..

[39] King A., Wooding P., Gardner L., Loke Y.W. (1993). Expression of Perforin, Granzyme A and TIA-1 by Human Uterine CD56+ NK Cells Implies They Are Activated and Capable of Effector Functions. Hum. Reprod..

[40] Rosental B., Brusilovsky M., Hadad U., Oz D., Appel M.Y., Afergan F., Yossef R., Rosenberg L.A., Aharoni A., Cerwenka A. (2011). Proliferating Cell Nuclear Antigen Is a Novel Inhibitory Ligand for the Natural Cytotoxicity Receptor NKp44 2011. J. Immunol..

[41] Kundu K., Ghosh S., Sarkar R., Edri A., Brusilovsky M., Gershoni-Yahalom O., Yossef R., Shemesh A., Soria J.-C., Lazar V. (2019). Inhibition of the NKp44-PCNA Immune Checkpoint Using a mAb to PCNA. Cancer Immunol. Res..

[42] Iraqi M., Edri A., Greenshpan Y., Goldstein O., Ofir N., Bolel P., Abu Ahmad M., Zektser M., Campbell K.S., Rouvio O. (2022). Blocking the PCNA/NKp44 Checkpoint to Stimulate NK Cell Responses to Multiple Myeloma. Int. J. Mol. Sci..

[43] Gaud G., Lesourne R., Love P.E. (2018). Regulatory Mechanisms in T Cell Receptor Signalling. Nat. Rev. Immunol..

[44] Kumar M., Michael S., Alvarado-Valverde J., Zeke A., Lazar T., Glavina J., Nagy-Kanta E., Donagh J.M., Kalman Z.E., Pascarelli S. (2024). ELM-the Eukaryotic Linear Motif Resource-2024 Update. Nucleic Acids Res..

[45] Macia E., Ehrlich M., Massol R., Boucrot E., Brunner C., Kirchhausen T. (2006). Dynasore, a Cell-Permeable Inhibitor of Dynamin. Dev. Cell.

[46] Wolf N.K., Kissiov D.U., Raulet D.H. (2023). Roles of Natural Killer Cells in Immunity to Cancer, and Applications to Immunotherapy. Nat. Rev. Immunol..

[47] Van den Herik-Oudijk I.E., Capel P.J., van der Bruggen T., Van de Winkel J.G. (1995). Identification of Signaling Motifs within Human Fc Gamma RIIa and Fc Gamma RIIb Isoforms. Blood.

[48] Owen D.J., Setiadi H., Evans P.R., McEver R.P., Green S.A. (2001). A Third Specificity-Determining Site in Mu 2 Adaptin for Sequences Upstream of Yxx Phi Sorting Motifs. Traffic.

[49] Goodridge J.P., Witt C.S., Christiansen F.T., Warren H.S. (2003). KIR2DL4 (CD158d) Genotype Influences Expression and Function in NK Cells. J. Immunol..

[50] Angata T., Tabuchi Y., Nakamura K., Nakamura M. (2007). Siglec-15: An Immune System Siglec Conserved throughout Vertebrate Evolution. Glycobiology.

[51] Kikuchi-Maki A., Yusa S., Catina T.L., Campbell K.S. (2003). KIR2DL4 Is an IL-2-Regulated NK Cell Receptor That Exhibits Limited Expression in Humans but Triggers Strong IFN-Gamma Production. J. Immunol..

[52] Cao H., Neerincx A., de Bono B., Lakner U., Huntington C., Elvin J., Gudgin E., Pridans C., Vickers M.A., Huntly B. (2021). Sialic Acid-Binding Immunoglobulin-like Lectin (Sigelac)-15 Is a Rapidly Internalised Cell-Surface Antigen Expressed by Acute Myeloid Leukaemia Cells. Br. J. Haematol..

[53] Barrow A.D., Cella M., Edeling M.A., Khan M.A.-A.-K., Cervantes-Barragan L., Bugatti M., Schmedt C., Vermi W., Colonna M. (2024). Cutting Edge: PDGF-DD Binding to NKp44 Costimulates TLR9 Signaling and Proinflammatory Cytokine Secretion in Human Plasmacytoid Dendritic Cells. J. Immunol..

[54] Bauer S., Kirschning C.J., Häcker H., Redecke V., Hausmann S., Akira S., Wagner H., Lipford G.B. (2001). Human TLR9 Confers Responsiveness to Bacterial DNA via Species-Specific CpG Motif Recognition. Proc. Natl. Acad. Sci. USA.

[55] Cella M., Otero K., Colonna M. (2010). Expansion of Human NK-22 Cells with IL-7, IL-2, and IL-1β Reveals Intrinsic Functional Plasticity. Proc. Natl. Acad. Sci. USA.

[56] Cella M., Fuchs A., Vermi W., Facchetti F., Otero K., Lennerz J.K., Doherty J.M., Mills J.C., Colonna M. (2009). A Human Natural Killer Cell Subset Provides an Innate Source of IL-22 for Mucosal Immunity. Nature.

[57] von Lilienfeld-Toal M., Nattermann J., Feldmann G., Sievers E., Frank S., Strehl J., Schmidt-Wolf I.G.H. (2006). Activated Γδ T Cells Express the Natural Cytotoxicity Receptor Natural Killer P44 and Show Cytotoxic Activity against Myeloma Cells. Clin. Exp. Immunol..

[58] Correia D.V., Fogli M., Hudspeth K., Da Silva M.G., Mavilio D., Silva-Santos B. (2011). Differentiation of Human Peripheral Blood Vδ1+ T Cells Expressing the Natural Cytotoxicity Receptor NKp30 for Recognition of Lymphoid Leukemia Cells. Blood.

[59] Meresse B., Curran S.A., Ciszewski C., Orbelyan G., Setty M., Bhagat G., Lee L., Tretiakova M., Semrad C., Kistner E. (2006). Reprogramming of CTLs into Natural Killer–like Cells in Celiac Disease. J. Exp. Med..

[60] Tang Q., Grzywacz B., Wang H., Kataria N., Cao Q., Wagner J.E., Blazar B.R., Miller J.S., Verneris M.R. (2008). Umbilical Cord Blood T Cells Express Multiple Natural Cytotoxicity Receptors after IL-15 Stimulation, but Only NKp30 Is Functional. J. Immunol..

