# Supplementary material for: The NKp44-1 Isoform Is an Activating Receptor for PDGF-DD Expressed on Natural Killer Cells
Source: Cancers (Basel). 2026 Mar 28;18(7):1099. doi: 10.3390/cancers18071099 (PMC13072323; doi:10.3390/cancers18071099)
Supplement: Supplementary file 1 [file cancers-18-01099-s001.zip › cancers-4184744-supplementary.pdf]

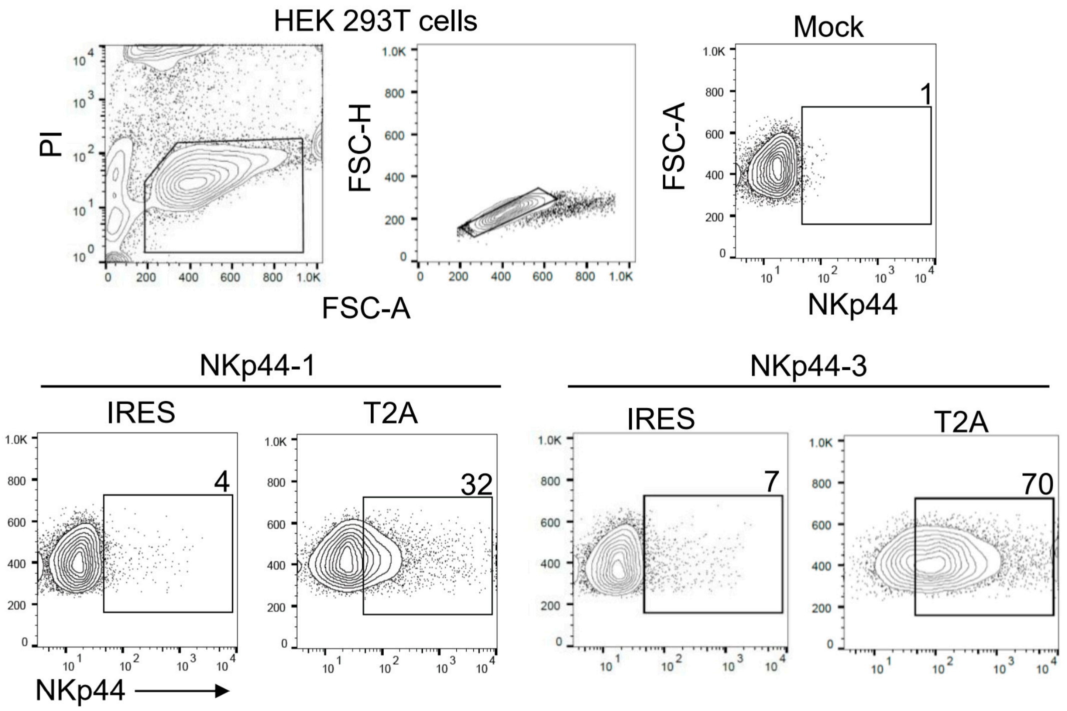

Figure S1. Surface expression of DAP12-T2A-NKp44 constructs in HEK 293T cells. NKp44-1 and NKp44-3 are detectable at the cell surface of HEK 293T cells transiently transfected with DAP12-T2A-NKp44, but not DAP12-IRES-NKp44, constructs. Representative dot plots depict NKp44⁺ (%) cells from two independent transfections stained in duplicate.

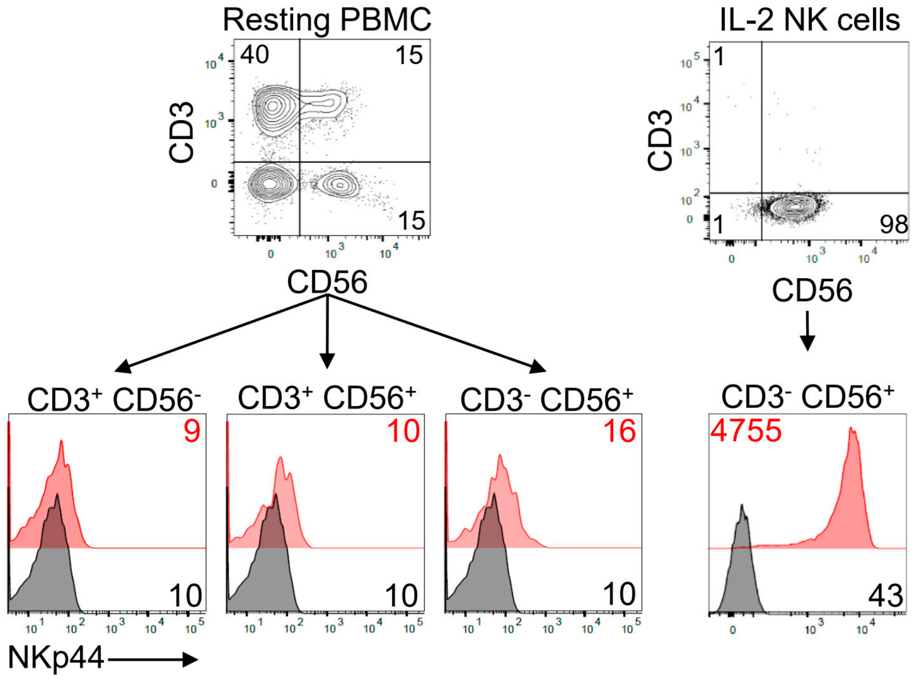

Figure S2. NK cell expression of NKp44 is inducible upon IL-2 activation. NKp44 expression is minimal in resting PBMCs but upregulated to the surface of $CD3^-$ $CD56^+$ NK cells in the presence of IL-2. Representative expression data for CD3, CD56 and NKp44 in resting PBMCs and IL-2 activated NK cells isolated from healthy donors used in this study.

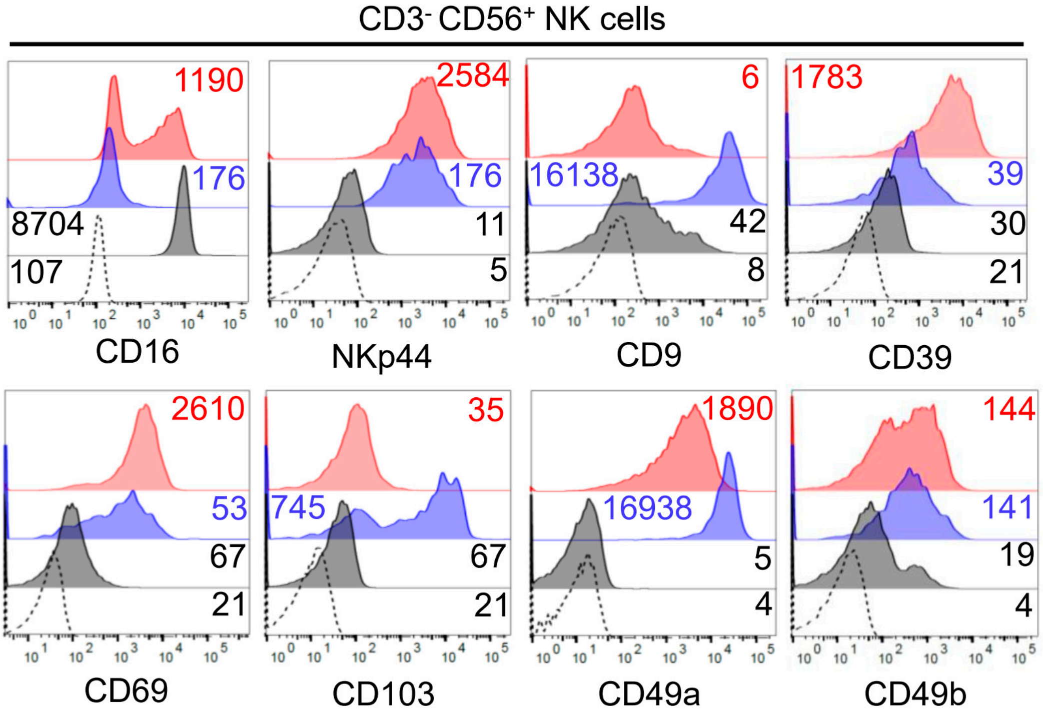

Figure S3 Expression of uterine NK cell markers in NK cells cultured in IL-2 with or without TGF-β. Surface expression of CD16, NKp44, CD9, CD39, CD69, CD103, CD49a and CD49b in $CD3^{-}$ $CD56^{+}$ freshly isolated NK cells (black histograms) or after culture in IL-2 (100 U/ml) alone (red histograms) or in combination with TGF-β (10 ng/ml) (blue histograms) for 14 days. Unstained freshly isolated NK cells were used as controls (dashed histograms). Representative histograms depict staining from one of two NK cell donors.

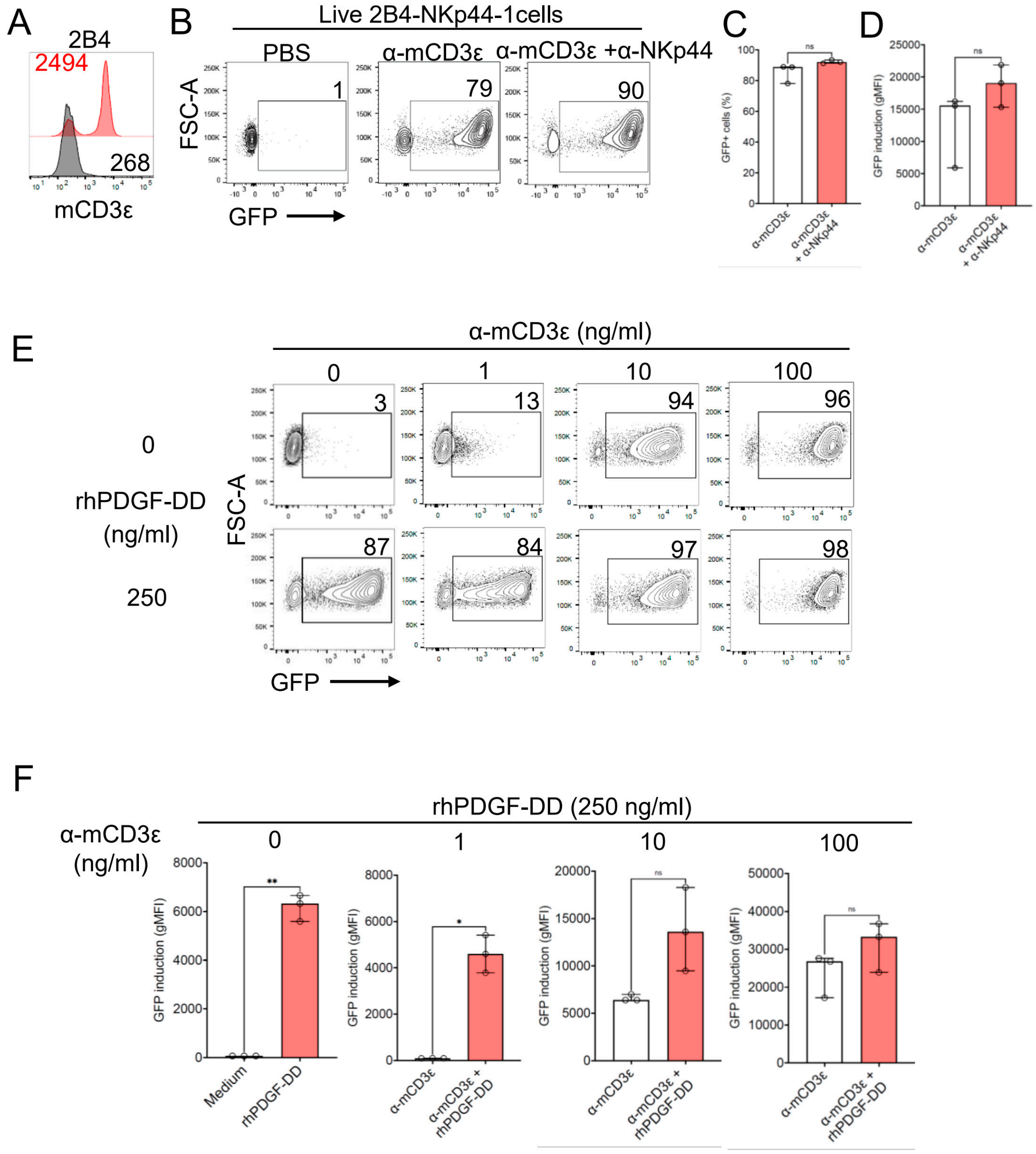

Figure S4 Stimulation of NKp44-1 enhances NFAT-GFP activation in response to mTCR agonism. A) 2B4 NFAT-GFP reporter cells endogenously express (red) mTCR as determined by anti-mCD3ε-PE staining, compared to (black) unstained cells. **B-D)** NFAT-GFP activation of 2B4-NKp44-1 cells after stimulation with either plate-bound anti-CD3e (10 μg/ml) alone or in combination with anti-NKp44 (10 μg/ml) for 16 hours, compared to PBS. Representative dot plots and column graphs depict median with IQR from three independent experiments performed in triplicate. **E)** GFP+ (%) and **F)** GFP signal intensity (gMFI) in 2B4-NKp44-1 cells stimulated with 250 ng/ml rhPDGF-DD in the presence of 0, 1, 10, or 100 ng/ml plate-bound anti-mCD3ε antibody for 16 hours. Representative dot plots and median with IQR gMFI data are representative of three independent experiments performed in triplicate. Statistical significance was determined using Welch's t tests. *p<0.05, **p<0.01.

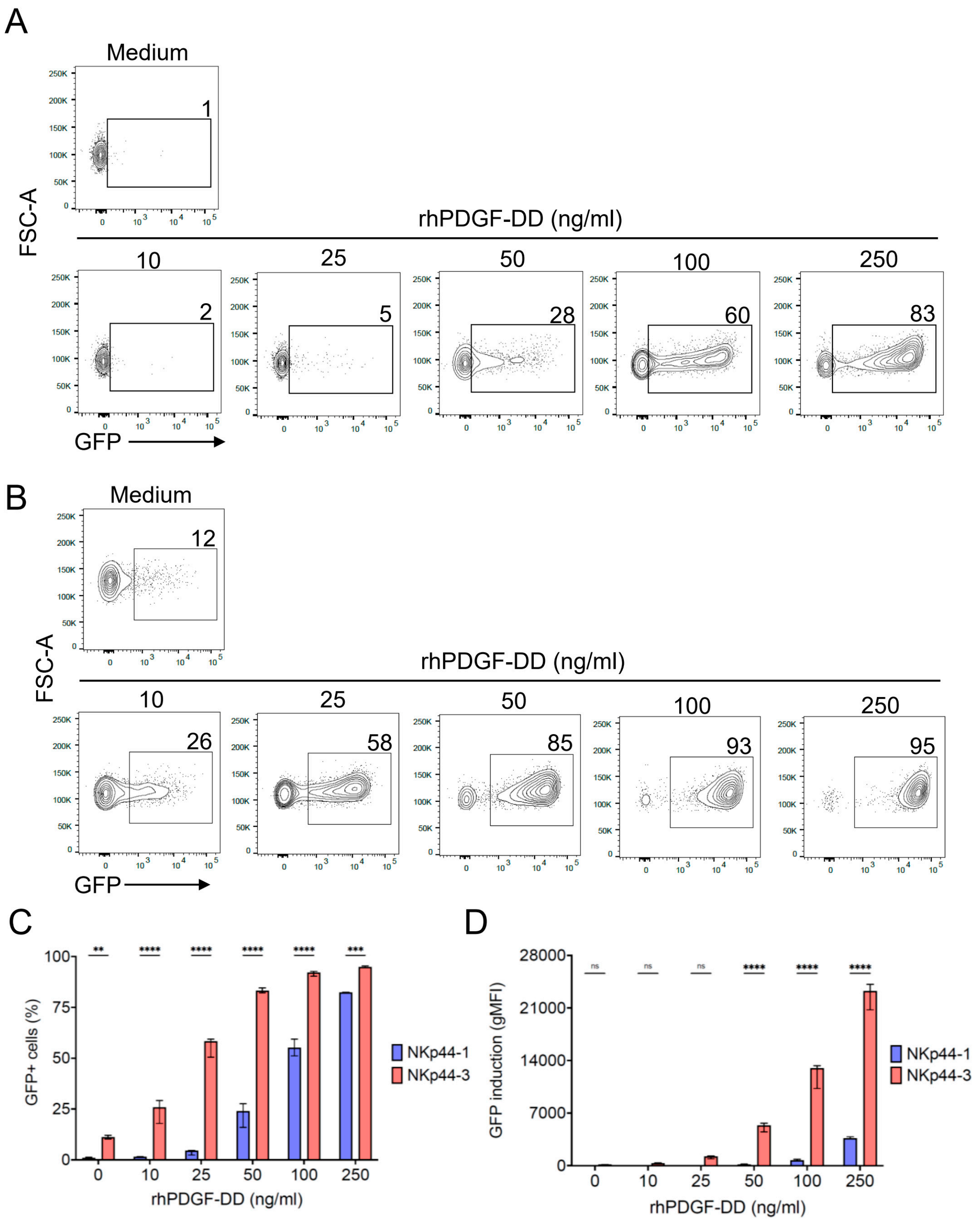

Figure S5 Differential activation response to free rhPDGF-D in polyclonal 2B4-NKp44-1 and 2B4-NKp44-3 NFAT-GFP reporter cells. Representative dot plots of GFP^{+} (%) **A)** 2B4-NKp44-1 and **B)** 2B4-NKp44-3 cells stimulated with 25, 50, 100 and 250 ng/ml rhPDGF-DD for 16 hours. Quantification of **C)** GFP+ cells (%) and **D)** mean intensity fluorescence of GFP in 2B4-NKp44-1 (blue) compared to 2B4-NKp44-3 cells (red) after 16 hours of stimulation with 0, 10, 25, 50, 100, or 250 ng/ml rhPDGF-DD. Representative dot plots and quantification depict median with IQR from three independent experiments performed in triplicate. Statistical significance was calculated using Šidák's multiple comparison test **p<0.01, ***p<0.001, ****p<0.0001.

Table S1 The predicted functional motifs embedded in the cytoplasmic domain of NKp44-1. The Eukaryotic Linear Motif Resource for Functional Sites in Proteins portal (http://elm.eu.org/) was searched for functional motifs in the cytoplasmic domain of NKp44-1. The NKp44-1 amino acid sequence (NKp44-1: NP_004819) was obtained from NCBI.

Instances (Matched Sequence)	Positions	Elm Description	
IA	1-2 [A]	N-terminal motif that initiates protein degradation by binding to the N-box of N-recognins. This N-degron variant comprises N-terminal a bulky hydrophobic residue as destabilizing residue.	2.30E-04
LVAKSLVLS	11-19 [A]	Both fungal and mammalian S-phase Cyclin/CDK complexes recognize specific RxL docking motifs in their target proteins.	1.88E-03
SVSSPV	58-63 [A]	The Class IV WW domain interaction motif is recognised primarily by the Pin1 phosphorylation-dependent prolyl isomerase.	1.54E-02
VPVFC	4-8 [A]	Binding motif for the N-lobe part of the C-terminal domain in Arc. The N-lobe domain has structural homology with HIV virus capsid, but the binding appears to be unique to higher vertebrates.	4.39E-03
DLPWTSVSSPV<br>EILYHTVARTKI	53-63 [A]<br>66-77 [A]	Canonical LIR motif that binds to Atg8/LC3 protein family members to mediate processes involved in autophagy.	3.63E-03
YHTVA	69-73 [A]	CRK family SH2 domain binding motif.	1.52E-03
WTSVSSP	56-62 [A]	This is the motif recognized by those SH3 domains with a non-canonical class I recognition specificity	1.32E-02
ILYHTV	67-72 [A]	ITIM (immunoreceptor tyrosine-based inhibitory motif). Phosphorylation of the ITIM motif, found in the cytoplasmic tail of some inhibitory receptors (KIRs) that bind MHC Class I, leads to the recruitment and activation of a protein tyrosine phosphatase.	2.99E-04
SVSSPVER	58-65 [A]	Longer version of the CDK phosphorylation site which shows specificity towards a lysine/arginine residue at position +4 after the phospho-Ser/Thr	1.93E-03
SLDTQKA<br>SVSSPVE	38-44 [A]<br>58-64 [A]	CK1 phosphorylation site	1.70E-02
WWKTMME<br>SVSSPVE	29-35 [A]<br>58-35 [A]	Casein kinase 2 (CK2) phosphorylation site	1.46E-02
VAKSLVLS<br>SLDTQKAT<br>LPWTSVSS<br>LYHTVART	12-19 [A]<br>38-45 [A]<br>54-61 [A]<br>68-75 [A]	GSK3 phosphorylation recognition site	2.68E-02
SLDTQKA	38-44 [A]	(ST)Q motif which is phosphorylated by PIKK family members.	9.23E-03
ELRSLDT	35-41 [A]	Ser/Thr residue phosphorylated by Plk2 and Plk3	2.18E-03
VAKSLVL<br>WWKTMME	12-18 [A]<br>29-35 [A]	Ser/Thr residue phosphorylated by Plk4	6.02E-03
SVSSPVE	58-64 [A]	Proline-Directed Kinase (e.g. MAPK) phosphorylation site in higher eukaryotes.	1.54E-02
YHTV	69-72 [A]	Tyrosine-based sorting signal responsible for the interaction with mu subunit of AP (Adaptor Protein) complex	2.59E-03
IALVPVFCGLLVA	1-13 [A]	Reverse NES binding the CRM1 groove in the minus direction. The spacing of the initial two hydrophobic residues ΦxΦ dictates the reverse orientation	1.19E-04

Table S2 The predicted functional motifs embedded in the cytoplasmic domain of NKp44-3. The Eukaryotic Linear Motif Resource for Functional Sites in Proteins portal (http://elm.eu.org/) was searched for functional motifs in the cytoplasmic domain of NKp44-3. The NKp44-3 amino acid sequence (NKp44-3: NP_001186439) was obtained from NCBI.

Instances (Matched Sequences)	Positions	Elm Description	
LVAKSLVLS	15-23 [A]	Both fungal and mammalian S-phase Cyclin/CDK complexes recognize specific RxL docking motifs in their target proteins.	1.88E-03
VPVFC	8-12 [A]	Binding motif for the N-lobe part of the C-terminal domain in Arc. The N-lobe domain has structural homology with HIV virus capsid, but the binding appears to be unique to higher vertebrates.	4.39E-03
LLHPAQP	43-49 [A]	This is the motif recognized by those SH3 domains with a non-canonical class I recognition specificity	1.32E-02
VAKSLVL	16-22 [A]	Ser/Thr residue phosphorylated by Plk4	6.02E-03
LRNR	31-34 [A]	The di-Arg ER retention motif is defined by two consecutive arginine residues (RR) or with a single residue insertion (RXR). The motif is completed by an adjacent hydrophobic/arginine residue which may be on either side of the Arg pair.	5.37E-03
IALVPVFCGLLVA	5-17 [A]	Reverse NES binding the CRM1 groove in the minus direction. The spacing of the initial two hydrophobic residues ΦxΦ dictates the reverse orientation	1.19E-04
